# Long-Term Modeling of SARS-CoV-2 Infection of *In Vitro* Cultured Polarized Human Airway Epithelium

**DOI:** 10.1128/mBio.02852-20

**Published:** 2020-11-06

**Authors:** Siyuan Hao, Kang Ning, Cagla Aksu Kuz, Kai Vorhies, Ziying Yan, Jianming Qiu

**Affiliations:** a Department of Microbiology, Molecular Genetics and Immunology, University of Kansas Medical Center, Kansas City, Kansas, USA; b Department of Anatomy and Cell Biology, University of Iowa, Iowa City, Iowa, USA; Virginia Polytechnic Institute and State University

**Keywords:** SARS-CoV-2, human airway epithelium, epithelial damage, recurrent infection, airway epithelial damage, long-term infection

## Abstract

Severe acute respiratory syndrome coronavirus 2 (SARS-CoV-2) replicates throughout human airways. The polarized human airway epithelium (HAE) cultured at an airway-liquid interface (HAE-ALI) is an *in vitro* model mimicking the *in vivo* human mucociliary airway epithelium and supports the replication of SARS-CoV-2. Prior studies characterized only short-period SARS-CoV-2 infection in HAE. In this study, continuously monitoring the SARS-CoV-2 infection in HAE-ALI cultures for a long period of up to 51 days revealed that SARS-CoV-2 infection was long lasting with recurrent replication peaks appearing between an interval of approximately 7 to 10 days, which was consistent in all the tested HAE-ALI cultures derived from 4 lung bronchi of independent donors. We also identified that SARS-CoV-2 does not infect HAE from the basolateral side, and the dominant SARS-CoV-2 permissive epithelial cells are ciliated cells and goblet cells, whereas virus replication in basal cells and club cells was not detected. Notably, virus infection immediately damaged the HAE, which is demonstrated by dispersed zonula occludens-1 (ZO-1) expression without clear tight junctions and partial loss of cilia. Importantly, we identified that SARS-CoV-2 productive infection of HAE requires a high viral load of >2.5 × 10^5^ virions per cm^2^ of epithelium. Thus, our studies highlight the importance of a high viral load and that epithelial renewal initiates and maintains a recurrent infection of HAE with SARS-CoV-2.

## Abstract (precis)

The pandemic of coronavirus disease 2019 (COVID-19), which is caused by severe acute respiratory syndrome coronavirus 2 (SARS-CoV-2), has led to >35 million confirmed cases and >1 million fatalities worldwide. SARS-CoV-2 mainly replicates in human airway epithelia in COVID-19 patients. In this study, we used *in vitro* cultures of polarized human bronchial airway epithelium to model SARS-CoV-2 replication for a period of 21 to 51 days. We discovered that *in vitro* airway epithelial cultures endure a long-lasting SARS-CoV-2 propagation with recurrent peaks of progeny virus release at an interval of approximately 7 to 10 days. Our study also revealed that SARS-CoV-2 infection causes airway epithelia damage with disruption of tight junction function and loss of cilia. Importantly, SARS-CoV-2 exhibits a polarity of infection in airway epithelium only from the apical membrane; it infects ciliated and goblet cells but not basal and club cells. Furthermore, the productive infection of SARS-CoV-2 requires a high viral load of over 2.5 × 10^5^ virions per cm^2^ of epithelium. Our study highlights that the proliferation of airway basal cells and regeneration of airway epithelium may contribute to the recurrent infections.

## INTRODUCTION

Coronavirus disease 2019 (COVID-19), an acute respiratory tract infection that emerged in late 2019, is caused by a novel coronavirus, severe acute respiratory syndrome coronavirus 2 (SARS-CoV-2) (1–5). It is an enveloped, positive-sense, single-stranded RNA virus, and belongs to the genus *Betacoronavirus* of the family *Coronaviridae* (1–3, 6). The clinical syndrome of COVID-19 is characterized by various degrees of severity, ranging from a mild upper respiratory illness (7) to severe interstitial pneumonia and acute respiratory distress syndrome (ARDS), a life-threatening lung injury that allows fluid to leak into the lung (5, 8–10). Compared to the approximately 34% fatality rate of the Middle East respiratory syndrome (MERS) and ∼10% fatality rate of the severe acute respiratory syndrome (SARS) (11), COVID-19 has a lower fatality rate, ranging from 0.7% to 5.7% in the United States (12); however, it spreads more efficiently than SARS and MERS (13, 14), making it difficult to contain. COVID-19 has become a pandemic (15), which has led to over 35.6 million confirmed cases and >1 million fatalities worldwide as of 5 October 2020.

While SARS-CoV-2 viral RNA can be detected in nasal swabs, nasopharyngeal aspirates, and bronchoalveolar lavage fluids throughout the airways (1–3, 16, 17), how the virus infects epithelial cells at different levels of the respiratory tree and the underlying pathogenesis remain unclear. Thus, a comprehensive understanding of how SARS-CoV-2 replicates and causes pathogenesis in its native host, the epithelial cells lining different levels of the airways, is essential to devising therapeutic and prevention strategies to counteract COVID-19. Primary human nasal, trachea, and bronchial epithelial cells can be cultured and differentiated at an air-liquid interface (ALI), forming a pseudostratified mucociliary airway epithelium that is composed of ciliated cells, goblet cells, club cells, and basal cells with an arrangement closely reflective of an *in vivo* cellular organization (18, 19). This *in vitro* model of human airway epithelium (HAE) cultured at an ALI (HAE-ALI) closely recapitulates many important characteristics of respiratory virus-host cell interactions seen in the infected upper and lower airways *in vivo* and has been used to study many human respiratory viruses (20–29), including SARS-CoV (30, 31). Primary HAE-ALI can be infected by SARS-CoV-2 (32, 33), resulting in epithelial damage (34), and they can be used for virus isolation (1, 16). Notably, differentiation at an ALI results in a drastic increase in expression and the polar presentation of the viral receptor angiotensin-converting enzyme 2 (ACE2) (2, 35) on the apical membrane (30, 31). Thus, HAE-ALI is an optimal cell culture model to study SARS-CoV-2 infection *in vitro*.

In this study, we generated HAE-ALI cultures directly from primary bronchial epithelial cells without propagation prior to differentiation at an ALI. We used these cultures to model SARS-CoV-2 infection for a long period of 21 to 51 days, focusing on the viral replication kinetics, the dose dependency of viral infection, epithelial damage, and the permissive subpopulation of the epithelial cell types. Additionally, we showed that SARS-CoV-2 favors apical infection of HAE-ALI, confirming the polar infection of SARS-CoV-2 in human airway epithelia. While SARS-CoV-2 efficiently infected HAE-ALI through the apical side at a viral load as low as a multiplicity of infection (MOI) of 0.002 plaque-forming units (PFU) per cell, viral replication at an MOI lower than this threshold was not detected. Notably, SARS-CoV-2 infection presented as an enduring infection in HAE-ALI with recurrent peaks of virus released from the infected ciliated and goblet cells, while the airway basal cells and club cells were nonpermissive.

## RESULTS

### SARS-CoV-2 infection of human airway epithelia presents a long-lasting infection and causes epithelial damage.

SARS-CoV-2 primarily infects human airway epithelial cells of the respiratory tracts and lungs of COVID-19 patients (36, 37). SARS-CoV-2 infections in the *in vitro* model of well-differentiated HAE-ALI and organoids have been reported (34, 36, 38, 39). However, these studies were focused on short-term virus replication and cytopathic effects as they were carried out in a time frame of less than 1 week postinfection. Since airway epithelia capably repair, regenerate, and remodel themselves (40), we hypothesized that a long-term monitoring of SARS-CoV-2 infection in HAE-ALI might reveal unknown important features that were missed in prior studies.

**FIG 1:**
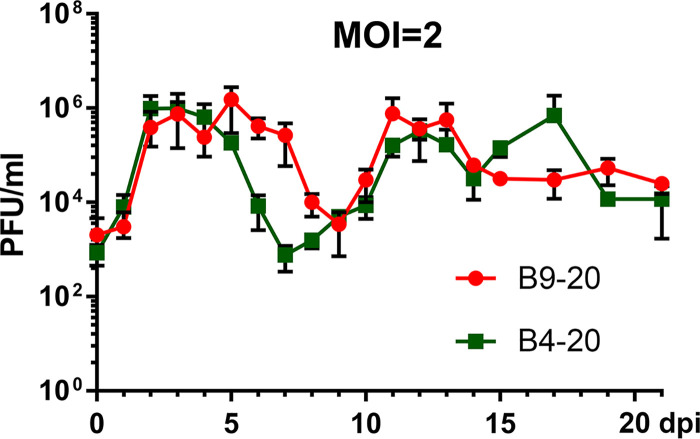
SARS-CoV-2 replication in primary human bronchial airway epithelium (HAE) over a course of 21 days. HAE-ALI^B4-20^ and HAE-ALI^B9-20^ cultures were infected with SARS-CoV-2 at an MOI of 2 from the apical side. At the indicated days postinfection (dpi), the apical surface was washed with 100 μl of D-PBS to collect the released virus. Plaque-forming units (PFU) were determined (*y* axis) and plotted to the day postinfection. Values represent means ± standard deviations (SD) (error bars).

To this end, we first chose two HAE-ALI cultures, B4-20 and B9-20 (HAE-ALI^B4-20^ and HAE-ALI^B9-20^), which were generated from primary bronchial epithelial cells freshly isolated from two donors. The initial study was performed with the infection of SARS-CoV-2 at an MOI of 2 PFU/cell. We collected the apical washes on a daily basis for continued monitoring of virus replication through titration for infectious virions with a plaque assay in Vero-E6 cells. We also periodically performed confocal microscopy analyses of the infected HAE with immunofluorescence assays. As expected, we observed rapid virus release from the infected HAE-ALI cultures, which reached a peak of 9 × 10^5^ PFU/ml at 2 days postinfection (dpi). The apical virus release from HAE-ALI^B4-20^ remained at the peak for 3 days and then decreased to a level less than 800 PFU/ml at 7 dpi (Fig. 1, B4-20). The infection of HAE-ALI^B9-20^ presented a similar trend, with the peak at 7.5 × 10^5^ PFU/ml from 2 to 6 dpi, which dropped to 3 × 10^3^ PFU/ml at 9 dpi (Fig. 1, B9-20). However, at later time points, continued study revealed virus release kinetics with at least two peaks during the course of 3 weeks from both infections. In the infection of HAE-ALI^B4-20^, virus release started to increase again from 8 dpi and reached a peak of 7.6 × 10^5^ PFU/ml at 12 dpi. The apical release of virus then dropped to 3 × 10^4^ PFU/ml at 14 dpi, followed by another peak of 7 × 10^5^ PFU/ml at 17 dpi (Fig. 1, B4-20). Notably, while the decrease of the virus released from the first peak of infected HAE-ALI^B9-20^ lagged behind that of the infected HAE-ALI^B4-20^ by 2 days, it demonstrated a second peak at 11 to 13 dpi (Fig. 1, B9-20) with viral shedding at 7.6 × 10^5^ PFU/ml. We reasoned that this was due to the donor variation, which affects the extent of differentiation and the subpopulation ratio of epithelial cell types, but not the properties permissive to SARS-CoV-2 infection.

**FIG 2:**
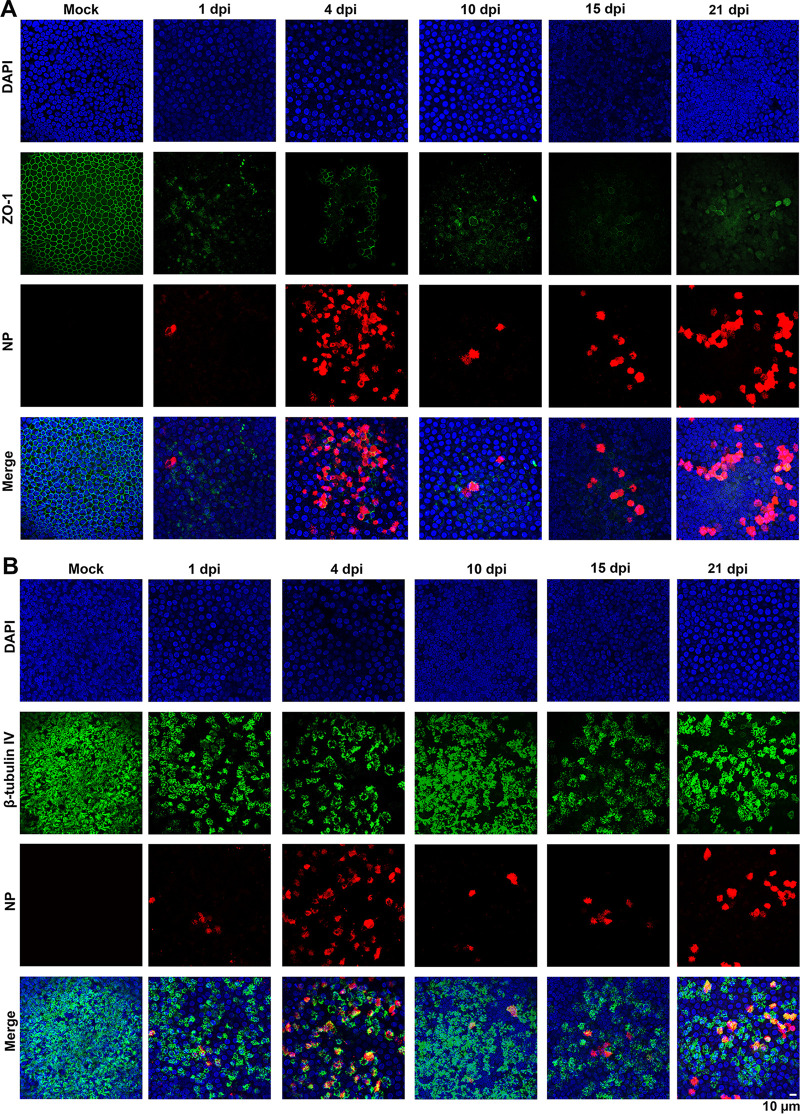
Immunofluorescence analysis of SARS-CoV-2-infected primary bronchial HAE-ALI over a course of 21 days. (A and B) Mock- and SARS-CoV-2-infected HAE-ALI^B4-20^ cultures were costained with anti-NP and anti-ZO-1 antibodies (A) or costained with anti-NP and anti-β-tubulin IV antibodies (B). Confocal images were taken at a magnification of ×40 on the indicated days postinfection (dpi). Nuclei were stained with DAPI (blue).

We next extended the study to another HAE-ALI culture derived from a different donor, the HAE-ALI^B3-20^. Infection was conducted with a 10-fold-reduced virus inoculum (MOI of 0.2 PFU/cell). We observed similar replication kinetics with two virus release peaks (see Fig. S1A in the supplemental material). Of note, even though a reduced MOI was applied to HAE-ALI^B3-20^, we recorded a higher viral shedding in the first peak (4 × 10^6^ PFU/ml) than that from HAE-ALI^B4-20^ and HAE-ALI^B9-20^, while those in the second peaks of the infections in the three cultures were approximately at a similar level (5 × 10^5^ PFU/ml). SARS-CoV-2 infection of HAE-ALI^B3-20^ significantly reduced the transepithelial electrical resistance (TEER) value, which is a hallmark of epithelial integrity, starting at 1 dpi (Fig. S1B) and resulted in dispersed zonula occludens-1 (ZO-1) expression and reduced β-tubulin IV staining that suggested the loss of cilia (Fig. S1C and D), which will be further discussed below. SARS-CoV2-infection in HAE-ALI was visualized by immunostaining for the expression of viral nucleocapsid protein (NP) in the infected cells. The analyses revealed the relative increases of NP-positive (NP+) cells, aligned roughly with the apical virus release kinetics (Fig. 2 and Fig. S2, NP). Of note, infected HAE-ALI showed poor staining of ZO-1, which started at 1 dpi and remained throughout the infection, indicating rapid epithelial damage caused by the infection as the tight junctions of the epithelia were destroyed (Fig. 2A, Fig. S1C, and Fig. S2A, ZO-1). The infected HAE-ALI also showed a partial loss of cilia, indicated by immunostaining with anti-β-tubulin IV, which also started at 1 dpi and remained at a similar level throughout the course of infection (Fig. 2B, Fig. S1D, and Fig. S2B).

10.1128/mBio.02852-20.1 FIG S1 Virus release kinetics and transepithelial electrical resistance measurement of HAE-ALI^B3-20^ infected with SARS-CoV-2 at an MOI of 0.2. (A) Virus release kinetics. The primary HAE^B3-20^ cultures were infected with SARS-CoV-2 at an MOI of 0.2 from the apical side. At the indicated days p.i. (dpi), 100 μl of apical washes by incubation of 100 μl of D-PBS in the apical chamber and 100 μl of the basolateral media were taken for plaque assays. Plaque-forming units (pfu) were plotted to the dpi. Values represent means ± standard deviations. (B) Change of TEER over the infection period. The TEER of mock- and SARS-CoV-2-infected HAE^B3-20^ cultures was measured using an epithelial volt-ohm meter (Millipore) at the indicated dpi and were normalized to the TEER measured at the first day, which is set at 1.0. Values represent means of the relative TEER ± standard deviations. ****, *P* < 0.0001. (C and D) Immunofluorescence analysis. Mock- and SARS-CoV-2-infected HAE-ALI^B3-20^ cultures at 5 and 21 dpi, respectively, were costained with anti-NP and anti-ZO-1 antibodies (C) or costained with anti-NP and anti-β-tubulin IV antibodies (D). Confocal images were taken at a magnification of ×40. Nuclei were stained with DAPI (blue). Download FIG S1, TIF file, 2.5 MB. Copyright © 2020 Hao et al. 2020 Hao et al. This content is distributed under the terms of the Creative Commons Attribution 4.0 International license.

10.1128/mBio.02852-20.2 FIG S2 Immunofluorescence analysis of SARS-CoV-2-infected HAE-ALI^B9-20^ at an MOI of 2 over a time course of 21 days. Mock- and SARS-CoV-2-infected HAE-ALI^B9-20^ cultures at the indicated days p.i. (dpi) were costained with anti-NP and anti-ZO-1 antibodies (A) or costained with anti-NP and anti-β-tubulin IV antibodies (B). Confocal images were taken at a magnification of ×40. Nuclei were stained with DAPI (blue). Download FIG S2, TIF file, 2.6 MB. Copyright © 2020 Hao et al. 2020 Hao et al. This content is distributed under the terms of the Creative Commons Attribution 4.0 International license.

**FIG 3:**
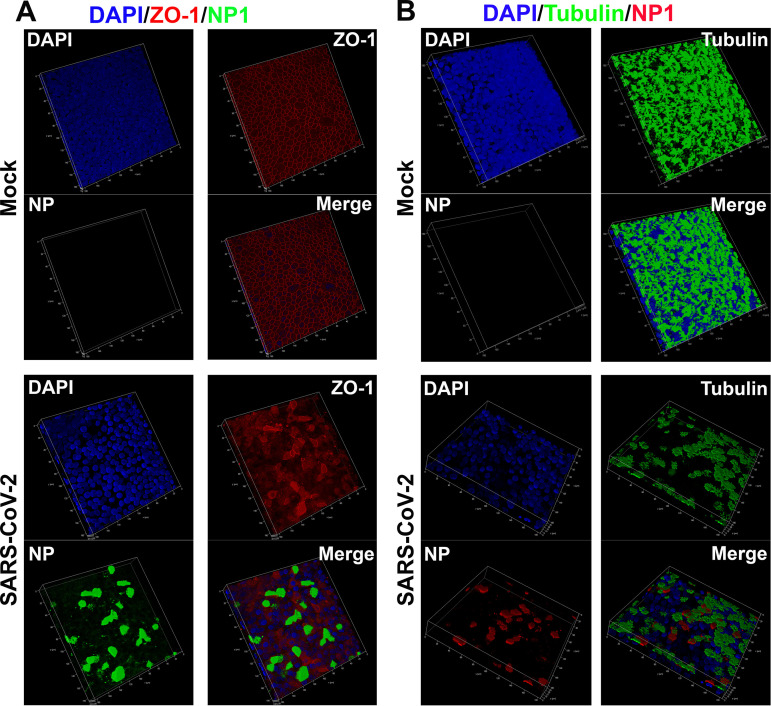
Three-dimensional (z-stack) imaging of SARS-CoV-2-infected primary bronchial HAE-ALI. (A and B) Mock- and SARS-CoV-2-infected HAE-ALI^B9-20^ cultures at 15 dpi were costained with anti-NP and anti-ZO-1 antibodies (A) or with anti-NP and anti-β-tubulin IV antibodies (B) or costained anti-NP and anti-ZO-1 antibodies (B). A set of confocal images were taken at a magnification of ×40 from the stained piece of epithelium at a distance of the Z value (in micrometers), shown in each image, from the objective (*z* axis) and reconstituted as a three-dimensional (z-stack) image as shown in each channel of fluorescence. Nuclei were stained with DAPI (blue).

To examine the infected epithelia in greater detail, we performed Z-stacked imaging of the infected HAE-ALI^B9-20^ at 15 dpi. The images showed a percentage of ∼10% NP+ cells, broken tight junctions (Fig. 3A, SARS-CoV-2/ZO-1), and an approximate loss of half of the cilia (Fig. 3B, SARS-CoV-2/Tubulin), compared with the mock-infected HAE (Fig. 3B, Mock). Of note, most of the NP+ cells remained β-tubulin IV stained, suggesting that ciliated cells represent the major cell type in HAE permissive to SARS-CoV-2. We also noticed that there were fewer cells present in the areas where NP1 staining was positive compared to the mock infection, as determined by the number of the nuclei in the imaged area, indicating cell loss (death) of the infected epithelia (Fig. 3, DAPI, SARS-CoV-2 versus Mock).

Taken all together, these results demonstrated that SARS-CoV-2 infection of HAE-ALI represents a long-lasting process with multiple peaks of virus infections (apical virus release and NP-expressing cells) and that the infection degrades two hallmarks of the airway epithelia, tight junctions and ciliary expression.

### SARS-CoV-2 infection of HAE presents multiple peaks and requires a high viral load.

**FIG 4:**
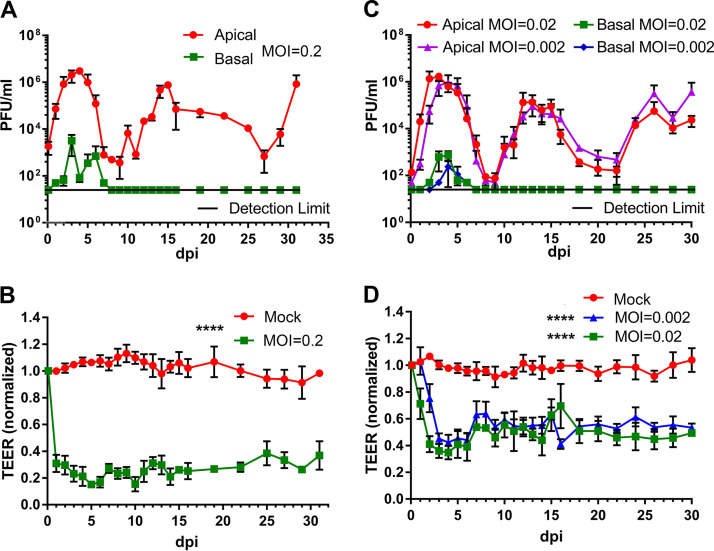
Virus release kinetics and transepithelial electrical resistance (TEER) measurement of HAE-ALI infected with SARS-CoV-2 at various viral loads (multiplicities of infection [MOIs]). (A and C) Virus release kinetics. HAE-ALI^B4-20^ cultures were infected with SARS-CoV-2 at an MOI of 0.2 (A) and 0.02 and 0.002 (C), respectively, from the apical side. At the indicated days postinfection (dpi), 100 μl of apical washes by incubation of 100 μl of D-PBS in the apical chamber and 100 μl of the basolateral media were taken for plaque assays. Plaque-forming units (PFU) were plotted to the dpi. Values represent means ± standard deviations. (B and D) TEER measurement. The TEER of mock- and SARS-CoV-2-infected HAE-ALI culture was measured using an epithelial volt-ohm meter (Millipore) at the indicated dpi. The TEER values were normalized to the TEER measured on the day of infection, which is set at 1.0. Values represent the means of relative TEER ± standard deviations. ****, *P* < 0.0001 by one-way Student’s *t* test.

**FIG 5:**
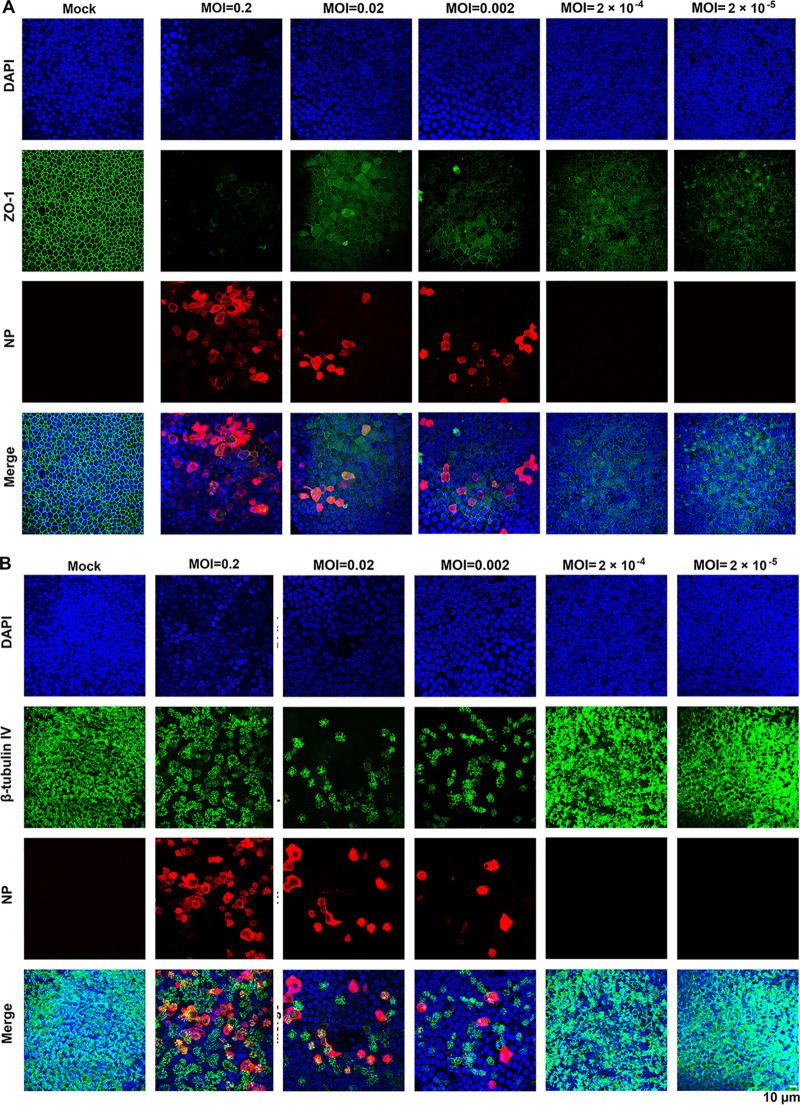
Immunofluorescence analysis of SARS-CoV-2-infected primary bronchial HAE at various viral loads (multiplicities of infection). HAE-ALI^B4-20^ cultures were infected with SARS-CoV-2 at an MOI from 0.2 to 2 × 10^−5^ PFU/cell. (A and B) At 30 dpi, both virus- and mock-infected HAE were costained with anti-NP and anti-ZO-1 antibodies (A) or costained with anti-NP and anti-β-tubulin IV antibodies (B). Confocal images were taken at a magnification of ×40. Nuclei were stained with DAPI (blue).

To further examine the recurrent peaks of virus release from the infections and the barrier dysfunction of SARS-CoV-2-infected HAE, we performed a longer monitoring period of 31 days for the infection of HAE-ALI^B4-20^ at an MOI from 0.2 to 2 × 10^−5^ (Fig. 4 and 5). We also infected HAE-ALI^L209^, which was polarized on the large MilliCell insert (area = 1.1 cm^2^), at an MOI of 0.2 and monitored the viral infection for a period of 51 days (Fig. S3 and S4). At an MOI of 0.2 PFU/cell, the infection of HAE-ALI^B4-20^ clearly displayed three peaks at 4, 15, and 31 dpi (Fig. 4A), and the infection of HAE-ALI^L209^ displayed five or six peaks at 3, 14, 23, 31, and 41 dpi (Fig. S3A). There were significant numbers of infected cells (NP+) at 31 and 51 dpi in HAE-ALI^B4-20^ and HAE-ALI^L209^, respectively (Fig. 5, MOI = 0.2, and Fig. S4). Again, the barrier function of the infected HAE-ALI was diminished, as determined by the TEER measurement, starting from 1 dpi (Fig. 4B and D and Fig. S3B), as well as by the dispersed ZO-1 staining (Fig. 5A, MOI = 0.2, and Fig. S4A). We also consistently observed drastic loss of the cilia (Fig. 5B, MOI = 0.2, and Fig. S4B).

10.1128/mBio.02852-20.3 FIG S3 Virus release kinetics and transepithelial electrical resistance measurement of HAE-ALI^L209^ infected with SARS-CoV-2 at an MOI of 0.2. (A) Virus release kinetics. The primary HAE-ALI^L209^ cultures were infected with SARS-CoV-2 at an MOI of 0.2 from the apical side. At the indicated days postinfection (dpi), 300 μl of apical washes by incubation of 300 μl of D-PBS in the apical chamber and 300 μl of the basolateral media were taken for plaque assays. Plaque-forming units (pfu) were plotted to the dpi. Values represent means ± standard deviations. (B) TEER measurement. The TEER of mock- and SARS-CoV-2-infected primary HAE-ALI^L209^ cultures was measured using an epithelial volt-ohm meter (Millipore) at the indicated dpi and were normalized to the TEER measured on the first day, which is set at 1.0. Values represent means of the relative TEER ± standard deviations. Download FIG S3, TIF file, 0.5 MB. Copyright © 2020 Hao et al. 2020 Hao et al. This content is distributed under the terms of the Creative Commons Attribution 4.0 International license.

10.1128/mBio.02852-20.4 FIG S4 Immunofluorescence analysis of SARS-CoV-2-infected HAE-ALI^L209^ at an MOI of 0.2. Mock- and SARS-CoV-2-infected HAE-ALI^L209^ cultures at 51 dpi were costained with anti-NP and anti-ZO-1 antibodies (A) or costained with anti-NP and anti-β-tubulin IV antibodies (B). Confocal images were taken at a magnification of ×40. Nuclei were stained with DAPI (blue). Download FIG S4, TIF file, 2.2 MB. Copyright © 2020 Hao et al. 2020 Hao et al. This content is distributed under the terms of the Creative Commons Attribution 4.0 International license.

Viral shedding to the culture medium in the basolateral chamber across the supportive membrane was also continuously monitored in the experiments. The infectious virions in the medium were detected in the early time points after the infection was initiated, but at a level of 2 to 3 orders of magnitude less than that in the apical washes (Fig. 4A, Fig. S1A, and Fig. S3A), suggesting that the progeny of the SARS-CoV-2 are predominately released from the apical membrane of the infected HAE. We concluded that the trace of detected viruses in the basolateral medium must come from the leakage of the apically secreted viruses across the supportive semipermeable membrane due to epithelial damage caused from SARS-CoV-2 infection. Interestingly, no infectious virions were found in the basal medium when the peaks of the viral progeny in the apical washes reappeared at the late time points, even though virus burdens were at similar levels at these peaks (Fig. 4A and C and Fig. S1A). These observations suggest the regeneration of the destructive mucosal lesions occurs during the SARS-CoV-2 infection and that such repair is sufficient to prevent the viral shedding to the basolateral chamber, although the repair did not lead to the recovery of the TEER of the infected HAE (Fig. 4B and D). A different observation came from the infection of HAE-ALI^L209^, in which the epithelial cells from donor L209 were cultured and polarized in the large Millicell inserts. Traces of viral shedding in the basal medium were also found at the late time points, and their levels changed responding to each peak of the virus replication (Fig. S3A). We reasoned that this is due to the inefficient epithelium repair in the infected HAE-ALI^L209^, which was affected by either donor variation or the different culture formats. We speculate that the repair of the airway epithelium through basal cell differentiation stops most potential leakage of the virus but is not sufficient to recover full airway function.

The recurrent peaks of virus progeny released during the course of infection were further displayed in infections of HAE-ALI^B4-20^ at lower MOIs of 0.02 and 0.002 (Fig. 4C), which show three almost identical peaks at 3, 13, and 26 dpi over the course of 30 days. Of note, the third peak became obvious at 26 dpi from these low-MOI infections compared to that at 30 dpi from the higher MOI of 0.2, but the amounts of infectious virions released from those peaks remained at roughly the same level (∼1 × 10^6^ PFU/ml). The epithelial damage was indicated by the decrease of TEER beginning at 2 and 3 dpi, respectively (Fig. 4D), as well as revealed by the dispersed ZO-1 expression and loss of cilia (Fig. 5A, MOIs = 0.02 and 0.002).

We then carried out the infection at the much lower MOIs of 2 × 10^−4^ and 2 × 10^−5^ over a course of 3 weeks. Surprisingly, we found that HAE-ALI^B4-20^ cultures were not productively infected by SARS-CoV-2, as evidenced by no NP+ cells at 30 dpi (Fig. 5, MOI = 2 × 10^−4^ and 2 × 10^−5^). No infectious virions released from the apical side in these low-MOI conditions were detectable. To verify this result, we performed infections in HAE-ALI^B9-20^ at MOIs of 2 × 10^−4^ and 2 × 10^−5^. The results reproduced the same observations of no productive infection in the cultures derived from a different lung donor (Fig. S5). This is in contrast to the SARS-CoV-2 infection in Vero-E6 cells. At MOIs of 2 × 10^−4^ and 2 × 10^−5^, we did not observe an obvious loss of cilia in both infected HAE-ALI^B4-20^ and HAE-ALI^B9-20^ (Fig. 5B and Fig. S5B); however, we observed a cytoplasmic expression and a weak junction expression of ZO-1 at 30 dpi (Fig. 5A) and 21 dpi (Fig. S5A) for infected HAE-ALI^B4-20^ and HAE-ALI^B9-20^, respectively. These results demonstrate that a high viral load (at least >100 PFU [∼8.2 × 10^4^ viral genome copies {vgc}]) to an epithelium of 0.33 cm^2^, which contains ∼5 × 10^5^ epithelial cells, is necessary to initiate a productive infection.

10.1128/mBio.02852-20.5 FIG S5 Immunofluorescence analysis of SARS-CoV-2-infected HAE-ALI^B9-20^ at MOIs of 2 × 10^−4^ and 2 × 10^−5^, respectively. Mock- and SARS-CoV-2-infected HAE-ALI^B9-20^ cultures at 21 dpi were costained with anti-NP and anti-ZO-1 antibodies (A) or costained with anti-NP and anti-β-tubulin IV antibodies (B). Confocal images were taken at a magnification of ×40. Nuclei were stained with DAPI (blue). Download FIG S5, TIF file, 2.2 MB. Copyright © 2020 Hao et al. 2020 Hao et al. This content is distributed under the terms of the Creative Commons Attribution 4.0 International license.

### Ciliated and goblet cells are permissive to SARS-CoV-2 but not the basal and club cells.

**FIG 6:**
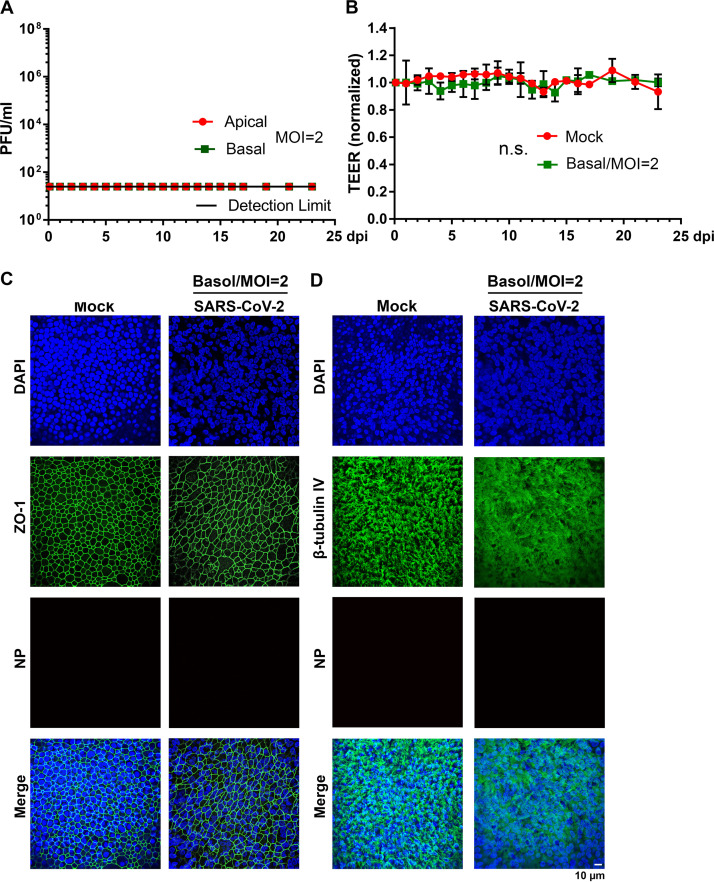
SARS-CoV-2 does not infect HAE-ALI from the basolateral side. (A) Virus release kinetics. Both apical washes and basolateral media were collected from SARS-CoV-2-infected HAE-ALI^B4-20^ every day and quantified for virus titers using plaque assays. Plaque-forming units (PFU) were plotted to the dpi. Value represent means ± standard deviations. (B) TEER measurement. The TEER of infected HAE-ALI^B4-20^ cultures was measured using an epithelial volt-ohm meter (Millipore) at the indicated dpi, and were normalized to the TEER measured on the first day, which is set at 1.0. Values represent means of the relative TEER ± standard deviations. n.s., statistically not significant. (C and D) Immunofluorescence analysis. Mock- and SARS-CoV-2-infected HAE-ALI^B4-20^ cultures at 23 dpi were costained with anti-NP and anti-ZO-1 antibodies (C) or costained with anti-NP and anti-β-tubulin IV antibodies (D). Confocal images were taken at a magnification of ×40. Nuclei were stained with DAPI (blue). Basol, basolateral.

We next examined SARS-CoV-2 infection in which a high MOI of 2 was applied to the basolateral side of HAE-ALI^B4-20^. The results showed there were no detectable infectious virions released from both the apical and basolateral sides (Fig. 6A). There were no signs of epithelial impairment observed as well. The TEER of the infected HAE displayed no significant changes over the course of 23 days (Fig. 6B). Immunofluorescence analyses revealed well-preserved tight junctions and the rich cilium expression (Fig. 6C and D). Importantly, NP+ cells were not detected for as long as 23 dpi. Similar results were verified in infection of HAE-ALI^B9-20^ over an infection course of 3 weeks. These results demonstrated that SARS-CoV-2 does not infect epithelial cells from the basolateral side.

**FIG 7:**
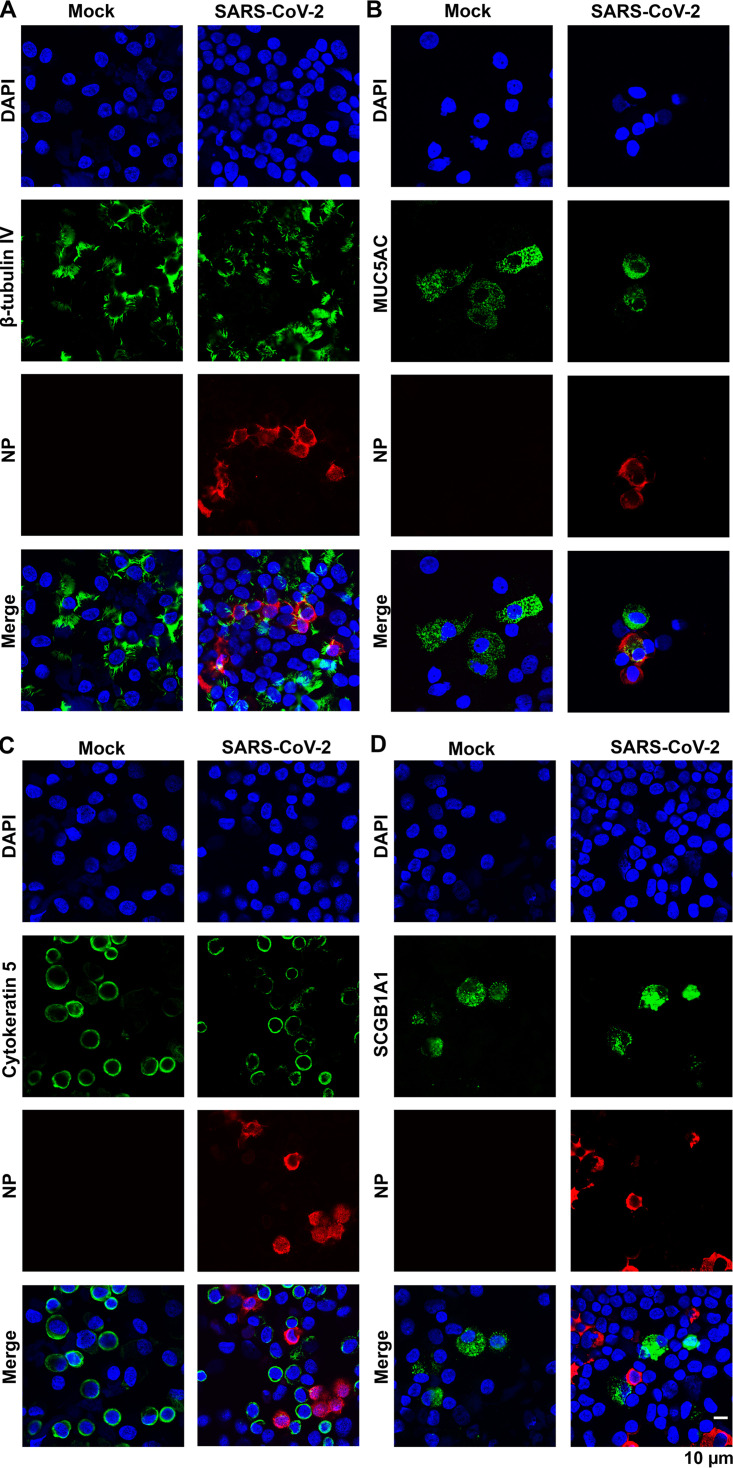
SARS-CoV-2 infects ciliated and goblet epithelial cells but not basal and club cells. Epithelial cells of the mock- and SARS-CoV-2-infected HAE-ALI^B9-20^ cultures at 4 dpi (MOI = 0.2) were dissociated from the Transwell insert and cytospun onto slides. The cells on the slides were fixed, permeabilized, and immunostained with anti-NP and together with anti-β-tubulin IV (A), and anti-MUC5AC (B), anti-cytokeratin 5 (C), and anti-SCGB1A1 (D), respectively. Confocal images were taken at a magnification of ×63. Nuclei were stained with DAPI (blue).

**FIG 8:**
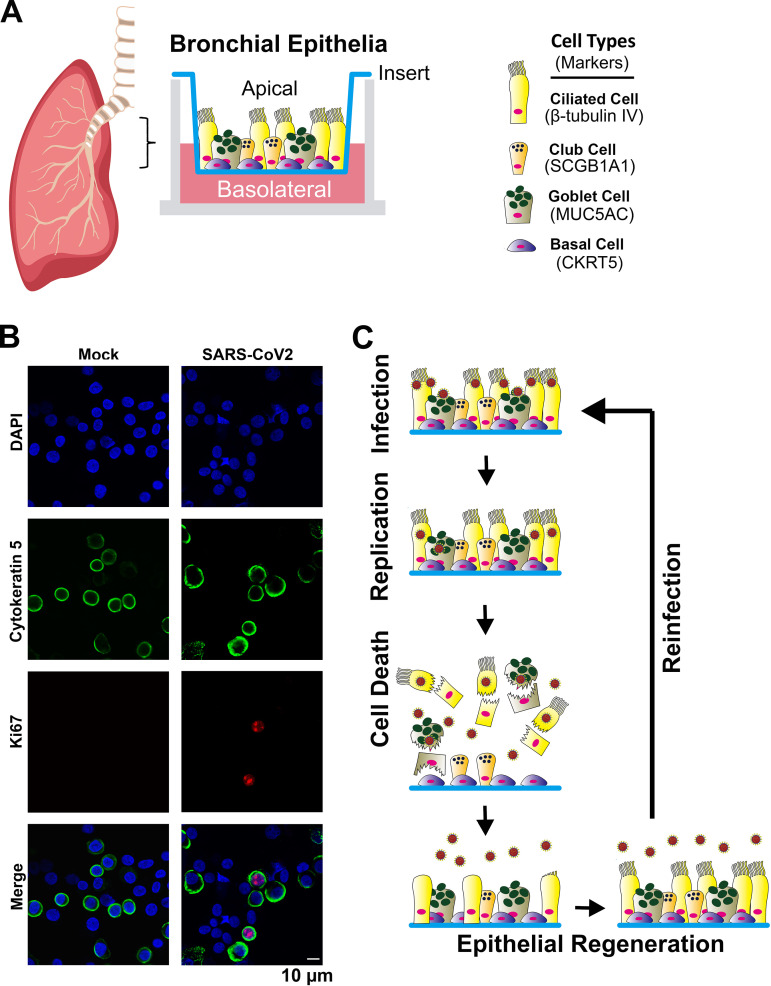
Diagram of HAE-ALI and model of the SARS-CoV-2 recurrent infection in HAE. (A) HAE-ALI model. Epithelial cells are taken from bronchia from the lungs of healthy donors and plated onto Transwell inserts at an air-liquid interface (ALI) for 4 weeks. Four major types of epithelial cells in the well-differentiated polarized HAE cultures, basal, ciliated, goblet, and club cells, are diagrammed in the Transwell insert, and their expression markers are indicated. (B) Basal cells in proliferation. Epithelial cells of the mock- and SARS-CoV-2-infected HAE-ALI^B9-20^ cultures at 9 dpi (MOI = 0.2) were dissociated from the Transwell insert and cytospun onto slides. The cells on the slides were fixed, permeabilized, and immunostained with anti-Ki67 and together with anti-CKRT5. Confocal images were taken at a magnification of ×63. Nuclei were stained with DAPI (blue). (C) Model of airway cell regeneration of SARS-CoV-2 recurrent infections. SARS-CoV-2 infects apical ciliated and goblet cells, where it replicates to produce infectious progeny and causes the death of the infected cells. The destructive lesion of epithelium induces basal cell proliferation and differentiation to regenerate ciliated and goblet cells, which are readily infected by SARS-CoV-2 in the next cycle of the recurrent infections.

To determine the permissive epithelial cell types in the infected HAE-ALI, we carefully examined the infected cells by immunofluorescence assays using various epithelial cell markers. Cells were dissociated from the supportive membranes of the infected Transwell inserts and cytospun onto slides for imaging. Costaining of a specific cell marker and the viral NP expression visualized the cell types permissive for SARS-CoV-2 infection. The results, as the representative images shown in Fig. 7, demonstrated that the majority of cell populations in the HAE-ALI were basal cells, which expressed cytokeratin 5 (CKRT5+) (41), and ciliated cells with positive anti-β-tubulin IV staining. Consistent with previous imaging results (Fig. 3), most of the NP+ cells were also positive with anti-β-tubulin IV staining (Fig. 7A), whereas almost all the CKRT5+ basal cells were negative for anti-NP staining (Fig. 7C). Probing secretoglobin family 1A member 1 (SCGB1A1) expression for club cells and mucin 5AC (MUC5AC) expression for goblet cells (41) revealed that the secretory cells were less abundant subpopulations in the infected HAE-ALI cultures. While we could not locate any club cells stained positively for NP expression (Fig. 7D), we found some NP+/MUC5AC+ goblet cells (Fig. 7B). Importantly, we observed that in SARS-CoV-2-infected HAE-ALI, a subset of CKRT5+ basal cells are found associated with the expression of Ki67, but not in mock-infected HAE-ALI (Fig. 8B). As Ki67 is a marker of cell proliferation (42), this result suggested that SARS-CoV-2 infection pushes basal cells toward proliferation.

Taking these lines of evidence together, our results confirm that SARS-CoV-2 mainly infects ciliated cells of HAE, as well as goblet cells, despite the lower abundance of goblet cells in HAE-ALI cultures. Our study suggests that basal and club cells are not permissive to SARS-CoV-2.

## DISCUSSION

In this study, we modeled SARS-CoV-2 infection in HAE-ALI cultures generated from primary bronchial epithelial cells directly isolated from 4 lungs of independent donors. The infections were conducted at various MOIs, from both apical and basolateral sides, and for a long period from 21 to 51 days. Our studies demonstrated that the SARS-CoV-2 infection of HAE results in multiple replication peaks of virus progeny at MOIs from 2 to as low as 0.002, although the length of peak emergence time varied from ∼7 to 10 days. The most striking result we obtained is the resistance of HAE to SARS-CoV-2 infection at an MOI of 2 × 10^−4^ PFU/cell (∼300 PFU/cm^2^ of epithelium), which is in contrast to the infection in Vero-E6 cells at the same MOI or lower. Our studies also revealed that the basal (CKRT5+) cells and club (SCGB1A1+) cells are not permissive, whereas ciliated cells (β-tubulin IV+) and goblet (MUC5AC+) cells form the primary body of permissive cells in the SARS-CoV-2-infected HAE.

Whole-mount immunohistochemistry of SARS-CoV-2-infected HAE cultures revealed that ciliated cells were the predominately infected cell type (38). With the single epithelial cell suspension recovered from the infected inserts, we prepared cytospin slides to investigate in detail the cell types permissive to the infection. It is curious that secretory goblet and club cells behave in opposite ways to SARS-CoV-2 infection, since both of these cell types are surface epithelial cells accessible on the airway lumen (38). The permissibility of goblet cells to SARS-CoV-2 infection was previously reported by immunochemistry staining of biopsy airway section specimens from COVID-19 patients (36), as well as being supported by a recent *in vitro* modeling of HAE (34). Club cells express the viral receptor ACE2, but at a lower level than goblet, ciliated, and basal cells (43) (see Fig. S6 in the supplemental material). Finding that both the club cells and the basal cells failed to establish productive SARS-CoV-2 infection suggests a possible lack of the expression of the entry essential transmembrane serine protease 2 (TMPRSS-2) in these cell types (44, 45).

10.1128/mBio.02852-20.6 FIG S6 Ciliated, basal, goblet, and club cells express ACE2. Epithelial cells of HAE-ALI^B9-20^ cultures were dissociated from the Transwell insert and cytospun onto slides. The cells on the slides were fixed, permeabilized, blocked, and immunostained with anti-ACE2 and together with anti-β-tubulin IV (A), anti-MUC5AC (B), anti-cytokeratin 5 (C), and anti-SCGB1A1 (D), respectively. Confocal images were taken at a magnification of ×63. Nuclei were stained with DAPI (blue). Download FIG S6, TIF file, 1.4 MB. Copyright © 2020 Hao et al. 2020 Hao et al. This content is distributed under the terms of the Creative Commons Attribution 4.0 International license.

We observed that SARS-CoV-2 was unable to infect epithelial cells from the basolateral side, where CKRT5+ basal cells reside. The airway basal cells are the epithelial cell type not presenting on the surface of the airway lumen; thus, they are not accessible to the virus on the apical side. However, when the infection commences and the epithelial damage occurs, the destructive mucosal lesions (and the death of the infected ciliated and goblet cells) would allow the virus to gain access to the basal cells (Fig. 8C). Indeed, the detectable virus shedding to the basolateral chamber indicates a possible window to expose the basal cells to SARS-CoV-2. Notably, these time points also represent the peaks of release of virus progeny. However, none of the CKRT5+ and NP+ cells were found in SARS-COV-2-infected HAE. The nonpermissive nature of basal cells to SARS-CoV-2 is likely due to the negligible expression of TMPRSS-2 (43), since it expresses ACE2 (Fig. S6C) (46, 47). Of note, we observed a subset of basal cells proliferating after SARS-CoV-2 infection, indicating that the basal cells should play an important role in repairing the epithelium lesions caused by viral infection.

At the airway epithelial cellular level, the tight-junction-associated proteins, such as ZO-1, occludin, and claudins, play a central part in the epithelial cytoprotection by maintaining a physical selective barrier between external and internal environments (40). The tight junction proteins are highly labile structures whose formation and structure may be very rapidly altered after airway injury, for example, airway inflammation. Proinflammatory cytokines have a drastic effect on tight junction expression and barrier functions, which significantly alter the epithelial barrier permeability (48–50). SARS-CoV-2 infection distorts the ZO-1 expression, and thereafter causes barrier dysfunction (TEER decrease). The infection not only alters the ZO-1 expression of infected (NP1+ cells) but also uninfected cells (NP-negative [NP−] cells) (Fig. 3). This is also true for the loss of cilia. We believe that SARS-CoV-2 infection produces inflammatory cytokines as an innate immunity response upon virus infection (51), which either disturbs ZO-1 and tubulin expression or alters their structures. The innate immunity response may also limit virus infection at the front line. In fact, SARS-CoV-2 requires a high viral load (>300 PFU/cm^2^ of HAE) to initiate a productive infection (Fig. 4). Of note, the infectious titer (PFU) was determined by plaque assay in Vero-E6 cells, which are interferon deficient (52). We determined that 1 PFU of SARS-CoV-2 in Vero-E6 cells has a particle (viral genome copy) number of 820, suggesting that a load of 2.46 × 10^5^ particles is required to productively infect 1 cm^2^ of the airway epithelium, which is much higher than the small DNA parvovirus human bocavirus 1 (HBoV1) we studied (53). HBoV1 can infect HAE at an MOI of as low as 0.001 genome copies per cell, which equals 1.5 × 10^3^ particles per 1 cm^2^ of the airway epithelium. Apparently, whether and how strong an innate immunity response is induced during SARS-CoV-2 infection of HAE-ALI cultures warrant further investigation.

Airway epithelium repair or regeneration is critical for the maintenance of the barrier function and the limitation of airway hyperreactivity (54). In a biopsy specimen study of fresh tracheas and lungs from five deceased COVID-19 patients, it was found that the epithelium was severely damaged in some parts of the trachea, and extensive basal cell proliferation was observed in the trachea, where ciliated cells were damaged, as well as in the intrapulmonary airways (37). These data support our conclusion that basal cells are not permissive to SARS-CoV-2. As a response to these previous findings, our study observed that a subset of proliferating basal cells in the SARS-CoV-2 infected HAE-ALI, but not in the mock-infected HAE-ALI (Fig. 8B). Thus, we hypothesize that SARS-CoV-2 infection induces basal cell proliferation, which accounts for the observed long-lasting infections with recurrent peaks of viral replication and warrants future investigation. In addition, we speculate that the apical virus release peaks might correlate with the progress of regeneration in the damaged epithelia, despite the finding that the recurrent infection of SARS-CoV-2 in newly differentiated permissive cells prevents the epithelium from being fully repaired, which was indicated by the dispersed ZO-1 expression over the course of infection.

Overall, we propose a model of SARS-CoV-2-infection of HAE (Fig. 8C): SARS-CoV-2 selectively infects ciliated and goblet cells on the surface of the airway lumen (the apical side of HAE). Upon invading these cells, SARS-CoV-2 replicates and produces infectious virions, which eventually leads to cell death and epithelial damage. Upon the destructive lesions, airway epithelium has the capacity to progressively repair and regenerate itself. Thus, basal cells (possibly also including club cells) proliferate and differentiate to ciliated cells or goblet cells to fill up the areas that have lost ciliated or goblet cells. Then, the virus released from the last round of infection infects newly regenerated ciliated or goblet cells (repaired epithelia), followed by the second round of active replication and virus production. Therefore, airway epithelial regeneration confers a persistent, cyclically peaked infection of SARS-CoV-2 in human epithelia.

## MATERIALS AND METHODS

### Ethics statement.

Primary human bronchial epithelial cells were isolated from the lungs of healthy human donors by the Cells and Tissue Core of the Center for Gene Therapy, University of Iowa, and the Department of Internal Medicine, University of Kansas Medical Center with the approval of the Institutional Review Board (IRB) of the University of Iowa and University of Kansas Medical Center, respectively.

### Cell line and virus.

**(i) Cell line.** Vero-E6 cells (ATCC CRL-1586) were cultured in Dulbecco’s modified Eagle’s medium (DMEM) (HyClone, catalog no. SH30022.01; GE Healthcare Life Sciences, Logan, UT) supplemented with 10% fetal bovine serum (FBS) (catalog no. F0926, Sigma, St. Louis, MO) at 37°C under a 5% CO_2_ atmosphere.

**(ii) Virus.** SARS-CoV-2 (NR-52281), isolate USA-WA1/2020, was obtained from BEI Resources, NIAID, NIH. The viruses were propagated in Vero-E6 cells once, titrated by plaque assay, aliquoted in Dulbecco’s phosphate-buffered saline (D-PBS) (pH 7.4), and stored at −80˚C. A biosafety protocol to work on SARS-CoV-2 in the biosafety level (BSL3) lab was approved by the Institutional Biosafety Committee of the University of Kansas Medical Center. The virus titer of the wild-type SARS-CoV-2 was 1.0 × 10^7^ PFU/ml, which equals a physical titer of 8.2 × 10^9^ viral genome copies (vgc)/ml determined by reverse transcription-quantitative PCR (RT-qPCR).

### Primary airway epithelium cultured at an air-liquid interface (HAE-ALI) **(Fig. 8A).**

The primary HAE-ALI cultures HAE-ALI^B3-20^, HAE-ALI^B4-20^, and HAE-ALI^B9-20^ were provided by the Cells and Tissue Core of the Center for Gene Therapy, University of Iowa (18, 55–57). These polarized HAE-ALI cultures were derived from three independent donors. The freshly isolated human bronchial epithelial cells from the donor tissues were seeded onto collagen-coated, semipermeable polycarbonate membrane inserts (0.33 cm^2^, 0.4-μm pore size, Costar Transwell, catalog no. 3413, Corning, New York), and grown at an ALI as previously described (58). The cultures were maintained in USG medium containing 2% Ultroser G (USG) serum substitute (Pall BioSepra, France). After 3 to 4 weeks of culture at an ALI, the polarized culture was fully differentiated. The polarity of the HAE was determined for the TEER using an epithelial volt-ohm meter (Millipore). A value of 1,000 Ω·cm^2^ or higher was chosen for SARS-CoV-2 infection as we previously used for HBoV1 infection (50, 59). HAE-ALI^L209^ cultures on 1.1 cm^2^ Millicell-PCF (Millipore, Billerica, MA) were provided by Dr. Matthias Salathe, which were generated following a published method (60) using primary airway bronchial epithelial cells isolated from the lung of a donor (L209).

### Virus infection, sample collection, and titration. (i) Virus infection.

For apical infection, well-differentiated primary HAE-ALI in Transwell inserts (0.33 cm^2^; Costar) or in Millicell inserts (1.1 cm^2^; Millipore) were inoculated with 100 μl or 300 μl of SARS-CoV-2 at various MOIs, as indicated in each figure legend, applied to the apical chamber. For basolateral infection, HAE-ALI cultures in Transwell inserts were inoculated with SARS-CoV-2 diluted in 500 μl of USG medium added to the basolateral chamber. The infected HAE-ALI cultures were incubated at 37°C and 5% CO_2_ for 1 h followed by aspiration of the virus from the apical or basolateral chamber and washing of the cells with D-PBS three times (the last wash was saved and used for plaque assay, which was presented as the virus residue right after infection at the day 0 postinfection [0 dpi]). The HAE-ALI cultures were then further cultured at 37°C and 5% CO_2_.

### (ii) Viral sample collection.

Viral samples were collected from both the apical wash of the epithelium surface and the culture medium in basolateral chamber at multiple time points. In brief, 100 μl (or 300 μl) of D-PBS was added to the apical chamber for a short incubation of 30 min at 37°C and 5% CO_2_. Thereafter, this apical wash was recovered carefully from the apical chamber without disturbing the culture. To quantitate the viruses released from the basal membrane to the culture medium, 100 μl of medium was collected from each basolateral chamber. The infectious titers in the collected samples were determined by plaque assays in Vero-E6 cells.

### (iii) Plaque assays.

Vero-E6 cells were seeded in 24-well plates at a density of ∼0.5 × 10^6^ cells and grown to confluence the second day. Virus (apical washes or basolateral media) was serially diluted 10-fold in D-PBS. Two hundred microliters of the diluent was added to each well and incubated for 1 h on a rocking rotator. After the virus diluent was removed, ∼0.5 ml of overlay medium (1% methylcellulose [Sigma, catalog no. M0387] in DMEM with 5% FBS) was added to each well. The plates were incubated at 37°C under 5% CO_2_ for 4 days. After the methylcellulose overlays were removed, the cells were fixed using the 10% formaldehyde solution for 30 min and stained with 1% crystal violet solution followed by extensive washing using distilled water. Plaques in each well were manually counted and multiplied by the dilution factor to determine the virus titer at the unit of plaque-forming units per milliliter.

### (iv) Reverse transcription and quantitative PCR (RT-qPCR).

To eliminate free viral RNA in the samples, 25 units of Benzonase (Sigma) was added to 100 μl of the virus samples for 30 min (61). The nuclease-treated samples were used for viral RNA extraction using the Viral RNA extraction kit (Quick-RNA Viral kit) (catalog no. R1035; Zymo Research) following the manufacturer’s instructions. Moloney murine leukemia virus (M-MLV) reverse transcriptase (catalog no. M368A; Promega) was used to reverse transcribe viral RNA with the reverse PCR primer according to the manufacturer’s instructions. A 2.5-μl portion of the cDNA was quantified by TaqMan qPCR in a reaction of 25 μl to determine the number of viral genome copies (vgc) using the CDC 2019-nCoV_N1 set of primers and probe, which were synthesized at IDT (Coralville, IA). The plasmid pcDNA6B-(SARS-CoV-2)N, which contains the SARS-CoV-2 NP gene (nucleotides [nt] 998 to 2244), was used as a reference control (1 vgc = 7 × 10^−12^ μg) to establish a standard curve for absolute quantification on an Applied Biosystems 7500 Fast system (Foster City, CA).

### Immunofluorescence confocal microscopy. (i) Immunofluorescence assay.

For analysis of the SARS-CoV-2 infection in the HAE grown on the supportive membranes of the Transwell inserts, we cut off the membranes from the inserts and fixed them with 4% paraformaldehyde in PBS at 4°C overnight. The fixed membrane was washed in PBS for 5 min three times and then split into 4 (for 0.33-cm^2^ membrane) or 8 (for 1.01-cm^2^ membrane) pieces for whole-mount immunostaining. For cell marker analysis, we dissociated the cells off the supportive membranes of the Transwell inserts by incubation with Accutase (Innovative Cell Technologies, Inc., San Diego, CA). After incubation for 1 h at 37°C, cells were completely detached from the membrane and well separated. Cells were collected and then cytocentrifuged at 1,800 rpm for 3 min onto slides using a Shandon Cytospin 3 cytocentrifuge. After the slides were cytospun, they were fixed overnight in 4% paraformaldehyde at 4°C.

The fixed HAE or dissociated cells were permeabilized with 0.2% Triton X-100 for 15 min at room temperature. Then, the slide was incubated with primary antibody in PBS with 2% FBS for 1 h at 37°C. After the membrane was washing, it was incubated with fluorescein isothiocyanate- and rhodamine-conjugated secondary antibodies, followed by staining of the nuclei with DAPI (4′,6-diamidino-2-phenylindole).

### (ii) Confocal microscopy.

The cells were then visualized using a Leica TCS SPE confocal microscope at the Confocal Core Facility of the University of Kansas Medical Center. Images were processed with the Leica Application Suite X software.

### Antibodies used.

Primary antibodies used were rabbit monoclonal anti-SARS-CoV-2 nucleocapsid (NP) (clone 001) (catalog no. 40143-R001; SinoBiological US, Wayne, PA) at a dilution of 1:25, mouse monoclonal anti-β-tubulin IV antibody (clone ONS.1A6) (catalog no. T7941; MilliporeSigma, St. Louis, MO) at 1:100, mouse anti-ZO-1 (clone 1/ZO-1) (catalog no. 610966; BD Bioscience, San Jose, CA) at 1:100, and rabbit anti-Ki67 (clone SP6) (ab1666; Abcam, Cambridge, MA) at 1:50. Mouse anti-MUC5AC (Santa Cruz Biotechnology, catalog no. sc-33667; 1:10), mouse anti-cytokeratin k5 (ThermoFisher Invitrogen, catalog no. MA5-12596; 1:50), rat anti-SCGB1A1 (ThermoFisher Invitrogen, catalog no. MAB4218; 1:50), and goat anti-ACE2 (Novus Biologicals, catalog no. AF933, 1:10) were used to mark epithelial cell types.

### Transepithelial electrical resistance (TEER).

One hundred microliters of D-PBS was added to the apical chamber to determine the TEER using a Millicell ERS-2 volt-ohm meter (MilliporeSigma, Burlington, MA) following a previously used method (58).

### Statistics.

Virus release kinetics were determined with the means and standard deviations obtained from at least three independent HAE-ALI^B3-20,^ HAE-ALI^B3-20^, and HAE-ALI^B9-20^ and from duplicated HAE-ALI^L209^ by using GraphPad Prism version 8.0. Error bars represent means and standard deviations (SD). Statistical significance (*P* value) was determined by using unpaired (Student) *t* test for comparison of two groups.

## References

[1] Zhu N, Zhang D, Wang W, Li X, Yang B, Song J, Zhao X, Huang B, Shi W, Lu R, Niu P, Zhan F, Ma X, Wang D, Xu W, Wu G, Gao GF, Tan W, China Novel Coronavirus Investigating and Research Team. 2020. A novel coronavirus from patients with pneumonia in China, 2019. N Engl J Med 382:727–733. doi:10.1056/NEJMoa2001017. 31978945 PMC7092803

[2] Zhou P, Yang XL, Wang XG, Hu B, Zhang L, Zhang W, Si HR, Zhu Y, Li B, Huang CL, Chen HD, Chen J, Luo Y, Guo H, Jiang RD, Liu MQ, Chen Y, Shen XR, Wang X, Zheng XS, Zhao K, Chen QJ, Deng F, Liu LL, Yan B, Zhan FX, Wang YY, Xiao GF, Shi ZL. 2020. A pneumonia outbreak associated with a new coronavirus of probable bat origin. Nature 579:270–273. doi:10.1038/s41586-020-2012-7. 32015507 PMC7095418

[3] Wu F, Zhao S, Yu B, Chen YM, Wang W, Song ZG, Hu Y, Tao ZW, Tian JH, Pei YY, Yuan ML, Zhang YL, Dai FH, Liu Y, Wang QM, Zheng JJ, Xu L, Holmes EC, Zhang YZ. 2020. A new coronavirus associated with human respiratory disease in China. Nature 579:265–269. doi:10.1038/s41586-020-2008-3. 32015508 PMC7094943

[4] Li Q, Guan X, Wu P, Wang X, Zhou L, Tong Y, Ren R, Leung KSM, Lau EHY, Wong JY, Xing X, Xiang N, Wu Y, Li C, Chen Q, Li D, Liu T, Zhao J, Liu M, Tu W, Chen C, Jin L, Yang R, Wang Q, Zhou S, Wang R, Liu H, Luo Y, Liu Y, Shao G, Li H, Tao Z, Yang Y, Deng Z, Liu B, Ma Z, Zhang Y, Shi G, Lam TTY, Wu JT, Gao GF, Cowling BJ, Yang B, Leung GM, Feng Z. 2020. Early transmission dynamics in Wuhan, China, of novel coronavirus-infected pneumonia. N Engl J Med 382:1199–1207. doi:10.1056/NEJMoa2001316. 31995857 PMC7121484

[5] Huang C, Wang Y, Li X, Ren L, Zhao J, Hu Y, Zhang L, Fan G, Xu J, Gu X, Cheng Z, Yu T, Xia J, Wei Y, Wu W, Xie X, Yin W, Li H, Liu M, Xiao Y, Gao H, Guo L, Xie J, Wang G, Jiang R, Gao Z, Jin Q, Wang J, Cao B. 2020. Clinical features of patients infected with 2019 novel coronavirus in Wuhan, China. Lancet 395:497–506. doi:10.1016/S0140-6736(20)30183-5. 31986264 PMC7159299

[6] Coronaviridae Study Group of the International Committee on Taxonomy of Viruses. 2020. The species Severe acute respiratory syndrome-related coronavirus: classifying 2019-nCoV and naming it SARS-CoV-2. Nat Microbiol 5:536–544. doi:10.1038/s41564-020-0695-z. 32123347 PMC7095448

[7] Wolfel R, Corman VM, Guggemos W, Seilmaier M, Zange S, Muller MA, Niemeyer D, Jones TC, Vollmar P, Rothe C, Hoelscher M, Bleicker T, Brunink S, Schneider J, Ehmann R, Zwirglmaier K, Drosten C, Wendtner C. 2020. Virological assessment of hospitalized patients with COVID-2019. Nature 581:465–469. doi:10.1038/s41586-020-2196-x. 32235945

[8] Chen N, Zhou M, Dong X, Qu J, Gong F, Han Y, Qiu Y, Wang J, Liu Y, Wei Y, Xia J, Yu T, Zhang X, Zhang L. 2020. Epidemiological and clinical characteristics of 99 cases of 2019 novel coronavirus pneumonia in Wuhan, China: a descriptive study. Lancet 395:507–513. doi:10.1016/S0140-6736(20)30211-7. 32007143 PMC7135076

[9] Wang D, Hu B, Hu C, Zhu F, Liu X, Zhang J, Wang B, Xiang H, Cheng Z, Xiong Y, Zhao Y, Li Y, Wang X, Peng Z. 2020. Clinical characteristics of 138 hospitalized patients with 2019 novel coronavirus-infected pneumonia in Wuhan, China. JAMA 323:1061–1069. doi:10.1001/jama.2020.1585. 32031570 PMC7042881

[10] Liu K, Fang YY, Deng Y, Liu W, Wang MF, Ma JP, Xiao W, Wang YN, Zhong MH, Li CH, Li GC, Liu HG. 2020. Clinical characteristics of novel coronavirus cases in tertiary hospitals in Hubei Province. Chin Med J (Engl) 133:1025–1031. doi:10.1097/CM9.0000000000000744. 32044814 PMC7147277

[11] Petrosillo N, Viceconte G, Ergonul O, Ippolito G, Petersen E. 2020. COVID-19, SARS and MERS: are they closely related? Clin Microbiol Infect 26:729–734. doi:10.1016/j.cmi.2020.03.026. 32234451 PMC7176926

[12] CDC COVID-19 Response Team. 2020. Geographic differences in COVID-19 cases, deaths, and incidence - United States, February 12-April 7, 2020. MMWR Morb Mortal Wkly Rep 69:465–471. doi:10.15585/mmwr.mm6915e4. 32298250 PMC7755058

[13] Sanche S, Lin YT, Xu C, Romero-Severson E, Hengartner N, Ke R. 2020. High contagiousness and rapid spread of severe acute respiratory syndrome coronavirus 2. Emerg Infect Dis 26:1470–1477. doi:10.3201/eid2607.200282. 32255761 PMC7323562

[14] Wiersinga WJ, Rhodes A, Cheng AC, Peacock SJ, Prescott HC. 2020. Pathophysiology, transmission, diagnosis, and treatment of coronavirus disease 2019 (COVID-19): a review. JAMA 324:782–793. doi:10.1001/jama.2020.12839.e4. 32648899

[15] Bedford J, Enria D, Giesecke J, Heymann DL, Ihekweazu C, Kobinger G, Lane HC, Memish Z, Oh MD, Sall AA, Schuchat A, Ungchusak K, Wieler LH, WHO Strategic and Technical Advisory Group for Infectious Hazards. 2020. COVID-19: towards controlling of a pandemic. Lancet 395:1015–1018. doi:10.1016/S0140-6736(20)30673-5. 32197103 PMC7270596

[16] Lu R, Zhao X, Li J, Niu P, Yang B, Wu H, Wang W, Song H, Huang B, Zhu N, Bi Y, Ma X, Zhan F, Wang L, Hu T, Zhou H, Hu Z, Zhou W, Zhao L, Chen J, Meng Y, Wang J, Lin Y, Yuan J, Xie Z, Ma J, Liu WJ, Wang D, Xu W, Holmes EC, Gao GF, Wu G, Chen W, Shi W, Tan W. 2020. Genomic characterisation and epidemiology of 2019 novel coronavirus: implications for virus origins and receptor binding. Lancet 395:565–574. doi:10.1016/S0140-6736(20)30251-8. 32007145 PMC7159086

[17] Rothe C, Schunk M, Sothmann P, Bretzel G, Froeschl G, Wallrauch C, Zimmer T, Thiel V, Janke C, Guggemos W, Seilmaier M, Drosten C, Vollmar P, Zwirglmaier K, Zange S, Wolfel R, Hoelscher M. 2020. Transmission of 2019-nCoV infection from an asymptomatic contact in Germany. N Engl J Med 382:970–971. doi:10.1056/NEJMc2001468. 32003551 PMC7120970

[18] Karp PH, Moninger TO, Weber SP, Nesselhauf TS, Launspach JL, Zabner J, Welsh MJ. 2002. An in vitro model of differentiated human airway epithelia. Methods for establishing primary cultures. Methods Mol Biol 188:115–137. doi:10.1385/1-59259-185-X:115. 11987537

[19] Fulcher ML, Gabriel S, Burns KA, Yankaskas JR, Randell SH. 2005. Well-differentiated human airway epithelial cell cultures. Methods Mol Med 107:183–206. doi:10.1385/1-59259-861-7:183. 15492373

[20] Pyrc K, Sims AC, Dijkman R, Jebbink M, Long C, Deming D, Donaldson E, Vabret A, Baric R, van der Hoek L, Pickles R. 2010. Culturing the unculturable: human coronavirus HKU1 infects, replicates, and produces progeny virions in human ciliated airway epithelial cell cultures. J Virol 84:11255–11263. doi:10.1128/JVI.00947-10. 20719951 PMC2953148

[21] Sims AC, Baric RS, Yount B, Burkett SE, Collins PL, Pickles RJ. 2005. Severe acute respiratory syndrome coronavirus infection of human ciliated airway epithelia: role of ciliated cells in viral spread in the conducting airways of the lungs. J Virol 79:15511–15524. doi:10.1128/JVI.79.24.15511-15524.2005. 16306622 PMC1316022

[22] Zhang L, Peeples ME, Boucher RC, Collins PL, Pickles RJ. 2002. Respiratory syncytial virus infection of human airway epithelial cells is polarized, specific to ciliated cells, and without obvious cytopathology. J Virol 76:5654–5666. doi:10.1128/jvi.76.11.5654-5666.2002. 11991994 PMC137037

[23] Palermo LM, Porotto M, Yokoyama CC, Palmer SG, Mungall BA, Greengard O, Niewiesk S, Moscona A. 2009. Human parainfluenza virus infection of the airway epithelium: viral hemagglutinin-neuraminidase regulates fusion protein activation and modulates infectivity. J Virol 83:6900–6908. doi:10.1128/JVI.00475-09. 19386708 PMC2698534

[24] Banach S, Orenstein JM, Fox LM, Randell SH, Rowley AH, Baker SC. 2009. Human airway epithelial cell culture to identify new respiratory viruses: coronavirus NL63 as a model. J Virol Methods 156:19–26. doi:10.1016/j.jviromet.2008.10.022. 19027037 PMC2671689

[25] Ayora-Talavera G, Shelton H, Scull MA, Ren J, Jones IM, Pickles RJ, Barclay WS. 2009. Mutations in H5N1 influenza virus hemagglutinin that confer binding to human tracheal airway epithelium. PLoS One 4:e7836. doi:10.1371/journal.pone.0007836. 19924306 PMC2775162

[26] Donaldson EF, Yount B, Sims AC, Burkett S, Pickles RJ, Baric RS. 2008. Systematic assembly of a full-length infectious clone of human coronavirus NL63. J Virol 82:11948–11957. doi:10.1128/JVI.01804-08. 18818320 PMC2583659

[27] Scull MA, Gillim-Ross L, Santos C, Roberts KL, Bordonali E, Subbarao K, Barclay WS, Pickles RJ. 2009. Avian influenza virus glycoproteins restrict virus replication and spread through human airway epithelium at temperatures of the proximal airways. PLoS Pathog 5:e1000424. doi:10.1371/journal.ppat.1000424. 19436701 PMC2673688

[28] Wang G, Deering C, Macke M, Shao J, Burns R, Blau DM, Holmes KV, Davidson BL, Perlman S, McCray PB, Jr. 2000. Human coronavirus 229E infects polarized airway epithelia from the apical surface. J Virol 74:9234–9239. doi:10.1128/jvi.74.19.9234-9239.2000. 10982370 PMC102122

[29] Hao W, Bernard K, Patel N, Ulbrandt N, Feng H, Svabek C, Wilson S, Stracener C, Wang K, Suzich J, Blair W, Zhu Q. 2012. Infection and propagation of human rhinovirus C in human airway epithelial cells. J Virol 86:13524–13532. doi:10.1128/JVI.02094-12. 23035218 PMC3503113

[30] Jia HP, Look DC, Shi L, Hickey M, Pewe L, Netland J, Farzan M, Wohlford-Lenane C, Perlman S, McCray PB, Jr. 2005. ACE2 receptor expression and severe acute respiratory syndrome coronavirus infection depend on differentiation of human airway epithelia. J Virol 79:14614–14621. doi:10.1128/JVI.79.23.14614-14621.2005. 16282461 PMC1287568

[31] Sims AC, Burkett SE, Yount B, Pickles RJ. 2008. SARS-CoV replication and pathogenesis in an in vitro model of the human conducting airway epithelium. Virus Res 133:33–44. doi:10.1016/j.virusres.2007.03.013. 17451829 PMC2384224

[32] Pizzorno A, Padey B, Julien T, Trouillet-Assant S, Traversier A, Errazuriz-Cerda E, Fouret J, Dubois J, Gaymard A, Lescure F-X, Dulière V, Brun P, Constant S, Poissy J, Lina B, Yazdanpanah Y, Terrier O, Rosa-Calatrava M. 2020. Characterization and treatment of SARS-CoV-2 in nasal and bronchial human airway epithelia. Cell Rep Med 1:100059. doi:10.1016/j.xcrm.2020.100059. 32835306 PMC7373044

[33] Sheahan TP, Sims AC, Zhou S, Graham RL, Pruijssers AJ, Agostini ML, Leist SR, Schafer A, Dinnon KH, III, Stevens LJ, Chappell JD, Lu X, Hughes TM, George AS, Hill CS, Montgomery SA, Brown AJ, Bluemling GR, Natchus MG, Saindane M, Kolykhalov AA, Painter G, Harcourt J, Tamin A, Thornburg NJ, Swanstrom R, Denison MR, Baric RS. 2020. An orally bioavailable broad-spectrum antiviral inhibits SARS-CoV-2 in human airway epithelial cell cultures and multiple coronaviruses in mice. Sci Transl Med 12:eabb5883. doi:10.1126/scitranslmed.abb5883. 32253226 PMC7164393

[34] Zhu N, Wang W, Liu Z, Liang C, Wang W, Ye F, Huang B, Zhao L, Wang H, Zhou W, Deng Y, Mao L, Su C, Qiang G, Jiang T, Zhao J, Wu G, Song J, Tan W. 2020. Morphogenesis and cytopathic effect of SARS-CoV-2 infection in human airway epithelial cells. Nat Commun 11:3910. doi:10.1038/s41467-020-17796-z. 32764693 PMC7413383

[35] Wrapp D, Wang N, Corbett KS, Goldsmith JA, Hsieh CL, Abiona O, Graham BS, McLellan JS. 2020. Cryo-EM structure of the 2019-nCoV spike in the prefusion conformation. Science 367:1260–1263. doi:10.1126/science.abb2507. 32075877 PMC7164637

[36] Hui KPY, Cheung MC, Perera RAPM, Ng KC, Bui CHT, Ho JCW, Ng MMT, Kuok DIT, Shih KC, Tsao SW, Poon LLM, Peiris M, Nicholls JM, Chan MCW. 2020. Tropism, replication competence, and innate immune responses of the coronavirus SARS-CoV-2 in human respiratory tract and conjunctiva: an analysis in ex-vivo and in-vitro cultures. Lancet Respir Med 8:687–695. doi:10.1016/S2213-2600(20)30193-4. 32386571 PMC7252187

[37] Fang Y, Liu H, Huang H, Li H, Saqi A, Qiang L, Que J. 2020. Distinct stem/progenitor cells proliferate to regenerate the trachea, intrapulmonary airways and alveoli in COVID-19 patients. Cell Res 30:705–707. doi:10.1038/s41422-020-0367-9. 32606347 PMC7325636

[38] Hou YJ, Okuda K, Edwards CE, Martinez DR, Asakura T, Dinnon KH, III, Kato T, Lee RE, Yount BL, Mascenik TM, Chen G, Olivier KN, Ghio A, Tse LV, Leist SR, Gralinski LE, Schäfer A, Dang H, Gilmore R, Nakano S, Sun L, Fulcher ML, Livraghi-Butrico A, Nicely NI, Cameron M, Cameron C, Kelvin DJ, de Silva A, Margolis DM, Markmann A, Bartelt L, Zumwalt R, Martinez FJ, Salvatore SP, Borczuk A, Tata PR, Sontake V, Kimple A, Jaspers I, O’Neal WK, Randell SH, Boucher RC, Baric RS. 2020. SARS-CoV-2 reverse genetics reveals a variable infection gradient in the respiratory tract. Cell 182:429–446. doi:10.1016/j.cell.2020.05.042. 32526206 PMC7250779

[39] Salahudeen AA, Choi SS, Rustagi A, Zhu J, de la O SM, Flynn RA, Margalef-Català M, Santos AJM, Ju J, Batish A, van Unen V, Usui VT, Zheng GXY, Edwards CE, Wagar LE, Luca V, Anchang B, Nagendran M, Nguyen K, Hart DJ, Terry JM, Belgrader P, Ziraldo SB, Mikkelsen TS, Harbury PB, Glenn JS, Garcia KC, Davis MM, Baric RS, Sabatti C, Amieva MR, Blish CA, Desai TJ, Kuo CJ. 2020. Progenitor identification and SARS-CoV-2 infection in long-term human distal lung organoid cultures. bioRxiv doi:10.1101/2020.07.27.212076. PMC8003326 33238290

[40] Puchelle E, Zahm JM, Tournier JM, Coraux C. 2006. Airway epithelial repair, regeneration, and remodeling after injury in chronic obstructive pulmonary disease. Proc Am Thorac Soc 3:726–733. doi:10.1513/pats.200605-126SF. 17065381

[41] Ruiz Garcia A, Deprez M, Lebrigand K, Cavard A, Paquet A, Arguel MJ, Magnone V, Truchi M, Caballero I, Leroy S, Marquette CH, Marcet B, Barbry P, Zaragosi LE. 2019. Novel dynamics of human mucociliary differentiation revealed by single-cell RNA sequencing of nasal epithelial cultures. Development 146:dev177428. doi:10.1242/dev.177428. 31558434 PMC6826037

[42] Scholzen T, Gerdes J. 2000. The Ki-67 protein: from the known and the unknown. J Cell Physiol 182:311–322. doi:10.1002/(SICI)1097-4652(200003)182:3<311::AID-JCP1>3.0.CO;2-9. 10653597

[43] Sungnak W, Huang N, Becavin C, Berg M, Queen R, Litvinukova M, Talavera-Lopez C, Maatz H, Reichart D, Sampaziotis F, Worlock KB, Yoshida M, Barnes JL, HCA Lung Biological Network. 2020. SARS-CoV-2 entry factors are highly expressed in nasal epithelial cells together with innate immune genes. Nat Med 26:681–687. doi:10.1038/s41591-020-0868-6. 32327758 PMC8637938

[44] Hoffmann M, Kleine-Weber H, Schroeder S, Kruger N, Herrler T, Erichsen S, Schiergens TS, Herrler G, Wu NH, Nitsche A, Muller MA, Drosten C, Pohlmann S. 2020. SARS-CoV-2 cell entry depends on ACE2 and TMPRSS2 and is blocked by a clinically proven protease inhibitor. Cell 181:271–280. doi:10.1016/j.cell.2020.02.052. 32142651 PMC7102627

[45] Zang R, Gomez Castro MF, McCune BT, Zeng Q, Rothlauf PW, Sonnek NM, Liu Z, Brulois KF, Wang X, Greenberg HB, Diamond MS, Ciorba MA, Whelan SPJ, Ding S. 2020. TMPRSS2 and TMPRSS4 promote SARS-CoV-2 infection of human small intestinal enterocytes. Sci Immunol 5:eabc3582. doi:10.1126/sciimmunol.abc3582. 32404436 PMC7285829

[46] Manna VJ, Caradonna SJ. 2020. The heterogeneous nature of the coronavirus receptor, angiotensin-converting enzyme 2 (ACE2) in differentiating airway epithelia. bioRxiv doi:10.1101/2020.07.09.190074. PMC8840828 35187520

[47] Wark PA, Pathinayake PS, Kaiko G, Nichol K, Ali A, Chen L, Sutanto EN, Garratt LW, Sohal SS, Lu W, Eapen MS, Oldmeadow C, Bartlett N, Reid A, Veerati P, Hsu AC-Y, Looi K, Iosifidis T, Stick SM, Hansbro PM, Kicic A. 2020. ACE2 expression is elevated in airway epithelial cells from aged and male donors but reduced in asthma. bioRxiv doi:10.1101/2020.07.26.20162248. PMC8014151 33455043

[48] Coyne CB, Gambling TM, Boucher RC, Carson JL, Johnson LG. 2003. Role of claudin interactions in airway tight junctional permeability. Am J Physiol Lung Cell Mol Physiol 285:L1166–L1178. doi:10.1152/ajplung.00182.2003. 12909588

[49] Huang Q, Deng X, Yan Z, Cheng F, Luo Y, Shen W, Lei-Butters DC, Chen AY, Li Y, Tang L, Söderlund-Venermo M, Engelhardt JF, Qiu J. 2012. Establishment of a reverse genetics system for studying human bocavirus in human airway epithelia. PLoS Pathog 8:e1002899. doi:10.1371/journal.ppat.1002899. 22956907 PMC3431310

[50] Deng X, Zou W, Xiong M, Wang Z, Engelhardt JF, Ye SQ, Yan Z, Qiu J. 2017. Human parvovirus infection of human airway epithelia induces pyroptotic cell death via inhibiting apoptosis. J Virol 91:e01533-17. doi:10.1128/JVI.01533-17. 29021400 PMC5709578

[51] Vanderheiden A, Ralfs P, Chirkova T, Upadhyay AA, Zimmerman MG, Bedoya S, Aoued H, Tharp GM, Pellegrini KL, Manfredi C, Sorscher E, Mainou B, Lobby JL, Kohlmeier JE, Lowen AC, Shi PY, Menachery VD, Anderson LJ, Grakoui A, Bosinger SE, Suthar MS. 2020. Type I and type III IFN restrict SARS-CoV-2 infection of human airway epithelial cultures. J Virol 94:e00985-20. doi:10.1128/JVI.00985-20. 32699094 PMC7495371

[52] Desmyter J, Melnick JL, Rawls WE. 1968. Defectiveness of interferon production and of rubella virus interference in a line of African green monkey kidney cells (Vero). J Virol 2:955–961. doi:10.1128/JVI.2.10.955-961.1968. 4302013 PMC375423

[53] Deng X, Yan Z, Luo Y, Xu J, Cheng Y, Li Y, Engelhardt J, Qiu J. 2013. In vitro modeling of human bocavirus 1 infection of polarized primary human airway epithelia. J Virol 87:4097–4102. doi:10.1128/JVI.03132-12. 23345515 PMC3624236

[54] McDowell EM, Becci PJ, Schürch W, Trump BF. 1979. The respiratory epithelium. VII. Epidermoid metaplasia of hamster tracheal epithelium during regeneration following mechanical injury. J Natl Cancer Inst 62:995–1008. 285300

[55] Zabner J, Karp P, Seiler M, Phillips SL, Mitchell CJ, Saavedra M, Welsh M, Klingelhutz AJ. 2003. Development of cystic fibrosis and noncystic fibrosis airway cell lines. Am J Physiol Lung Cell Mol Physiol 284:L844–L854. doi:10.1152/ajplung.00355.2002. 12676769

[56] Yan Z, Zak R, Zhang Y, Ding W, Godwin S, Munson K, Peluso R, Engelhardt JF. 2004. Distinct classes of proteasome-modulating agents cooperatively augment recombinant adeno-associated virus type 2 and type 5-mediated transduction from the apical surfaces of human airway epithelia. J Virol 78:2863–2874. doi:10.1128/jvi.78.6.2863-2874.2004. 14990705 PMC353734

[57] Yan Z, Lei-Butters DC, Liu X, Zhang Y, Zhang L, Luo M, Zak R, Engelhardt JF. 2006. Unique biologic properties of recombinant AAV1 transduction in polarized human airway epithelia. J Biol Chem 281:29684–29692. doi:10.1074/jbc.M604099200. 16899463 PMC1712671

[58] Yan Z, Deng X, Qiu J. 2020. Human bocavirus 1 infection of well-differentiated human airway epithelium. Curr Protoc Microbiol 58:e107. doi:10.1002/cpmc.107. 32639683 PMC7422954

[59] Deng X, Yan Z, Cheng F, Engelhardt JF, Qiu J. 2016. Replication of an autonomous human parvovirus in non-dividing human airway epithelium is facilitated through the DNA damage and repair pathways. PLoS Pathog 12:e1005399. doi:10.1371/journal.ppat.1005399. 26765330 PMC4713420

[60] Schmid A, Bai G, Schmid N, Zaccolo M, Ostrowski LE, Conner GE, Fregien N, Salathe M. 2006. Real-time analysis of cAMP-mediated regulation of ciliary motility in single primary human airway epithelial cells. J Cell Sci 119:4176–4186. doi:10.1242/jcs.03181. 16984973

[61] Hao S, Ning K, Wang X, Wang J, Cheng F, Ganaie SS, Tavis JE, Qiu J. 2020. Establishment of a replicon system of the emerging tick-borne Bourbon Virus and use it for evaluation of antivirals. Front Microbiol 11:572631. doi:10.3389/fmicb.2020.572631. 33013808 PMC7506111

