# Supplementary figures and images for: Long-Term Modeling of SARS-CoV-2 Infection of In Vitro Cultured Polarized Human Airway Epithelium
Source: mBio. 2020 Nov 6;11(6):e02852-20. doi: 10.1128/mBio.02852-20 (PMC7649230; doi:10.1128/mBio.02852-20)

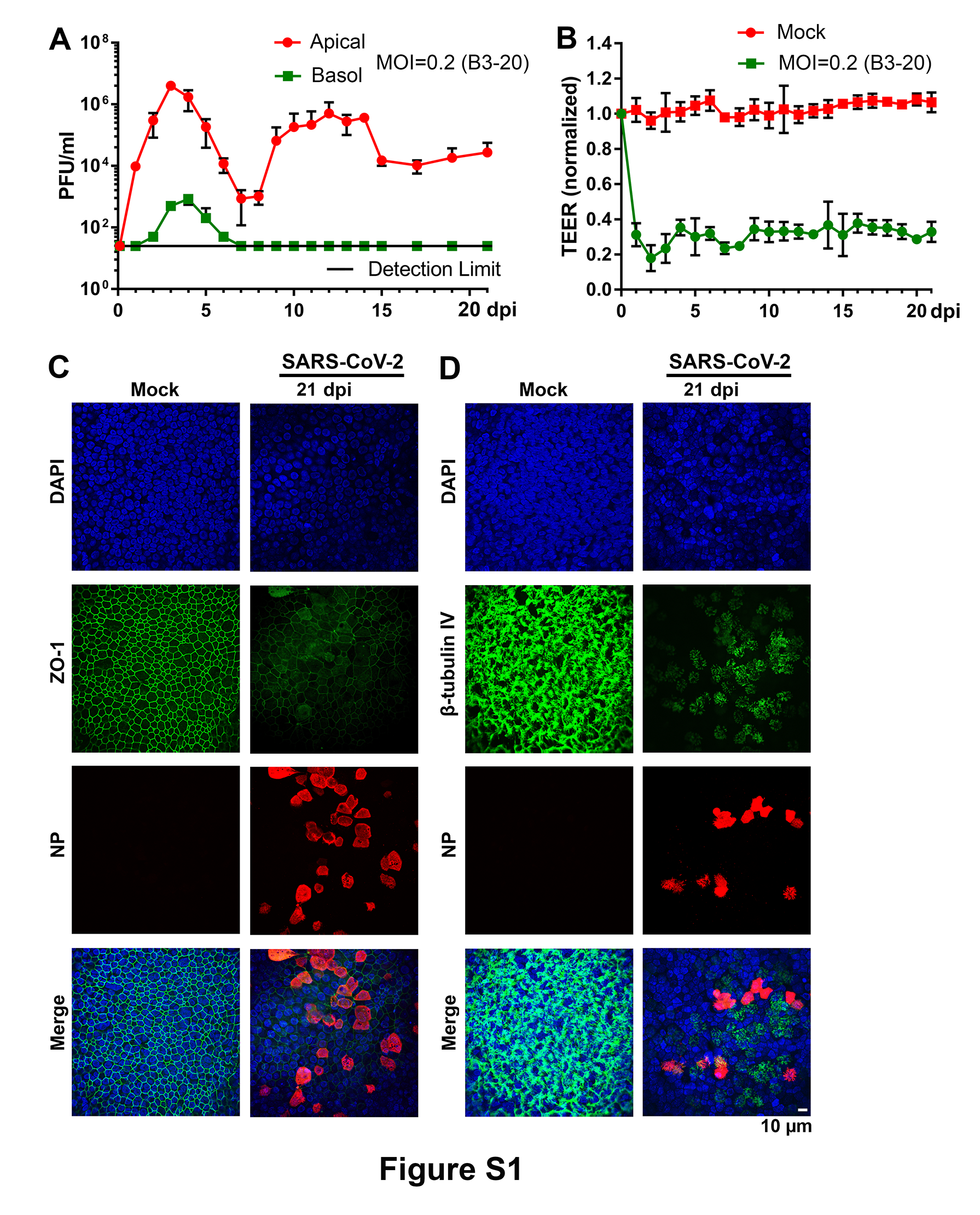

Supplement: FIG S1 [file mBio.02852-20-sf001.tif]

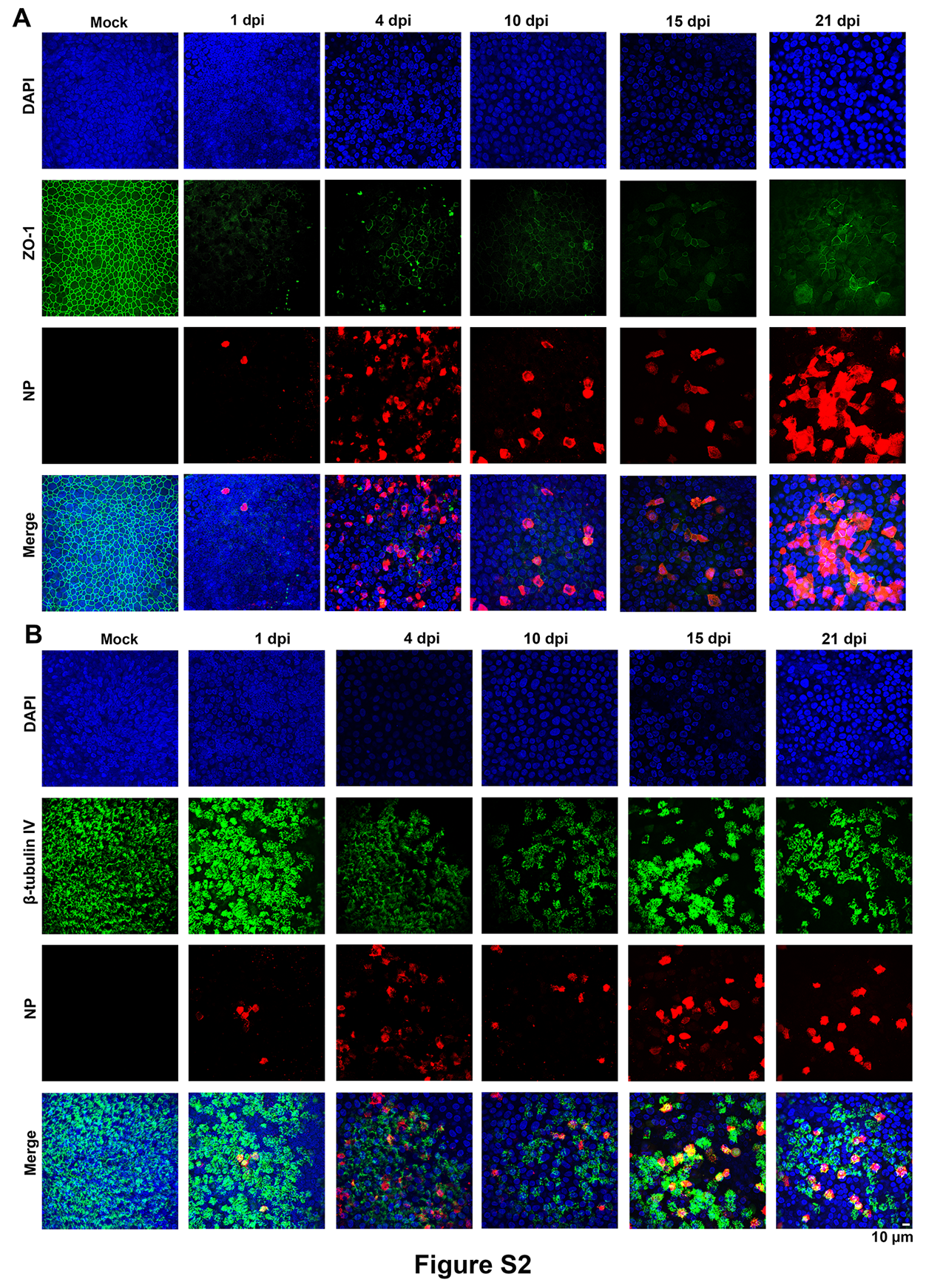

Supplement: FIG S2 [file mBio.02852-20-sf002.tif]

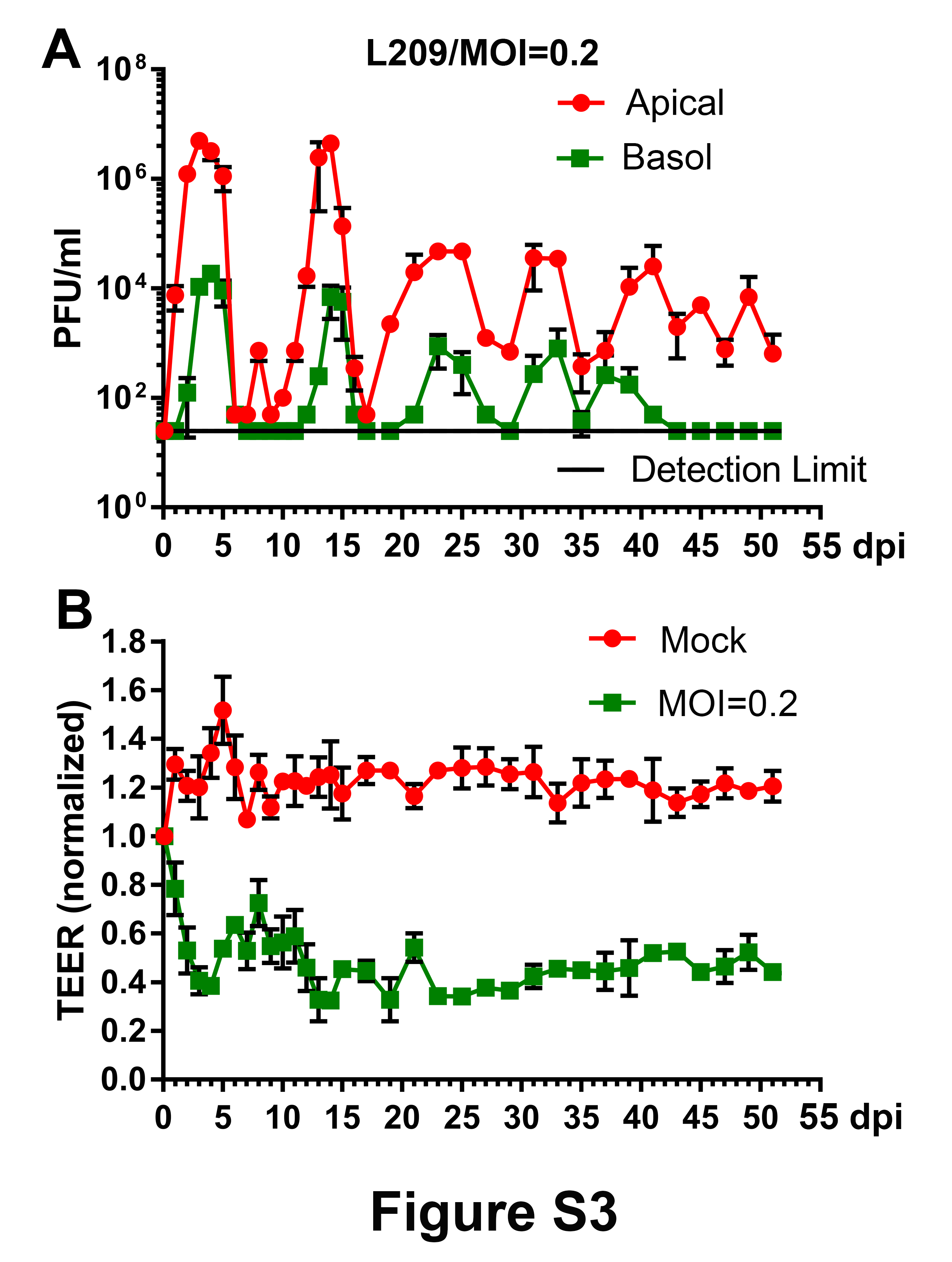

Supplement: FIG S3 [file mBio.02852-20-sf003.tif]

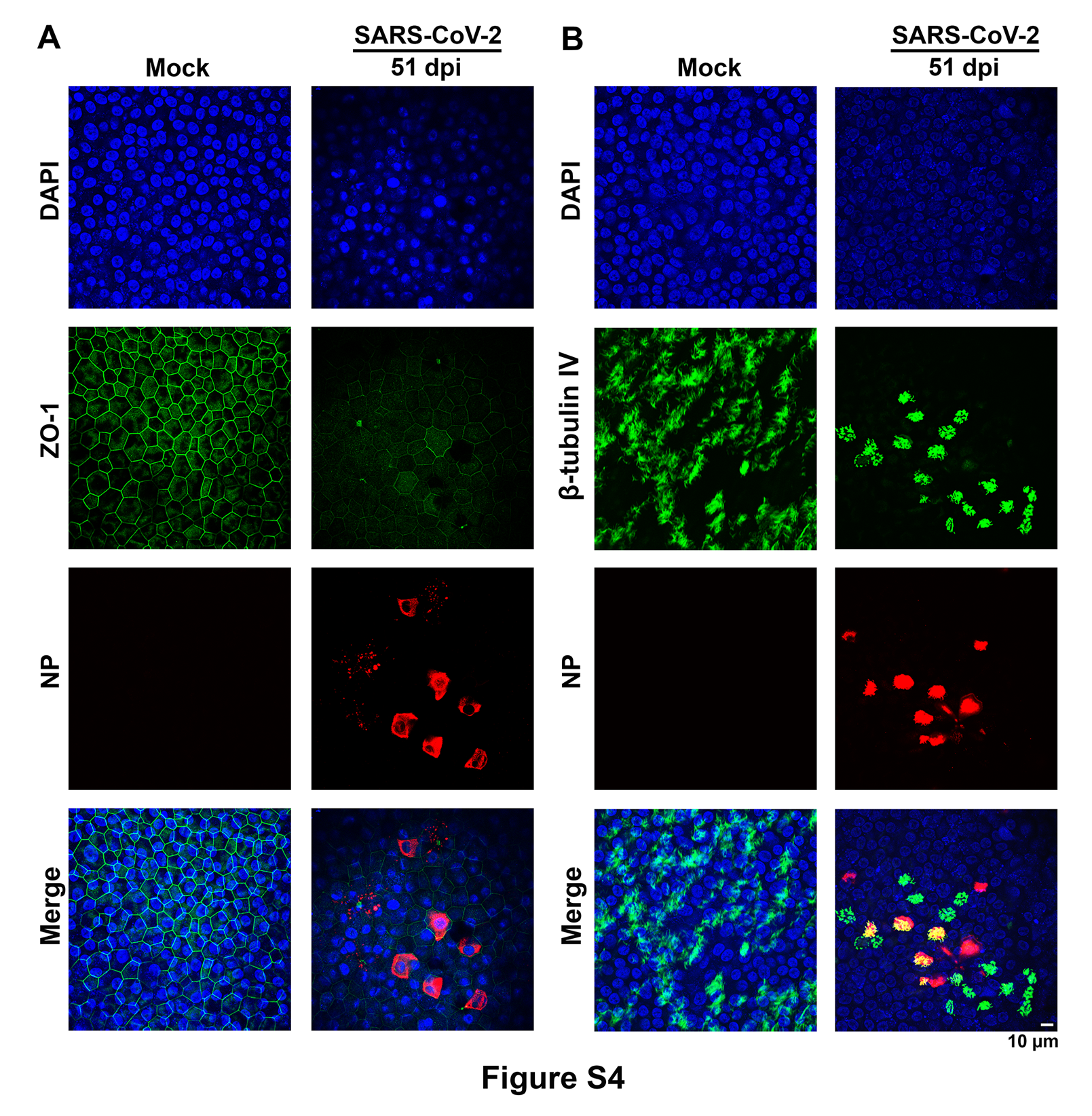

Supplement: FIG S4 [file mBio.02852-20-sf004.tif]

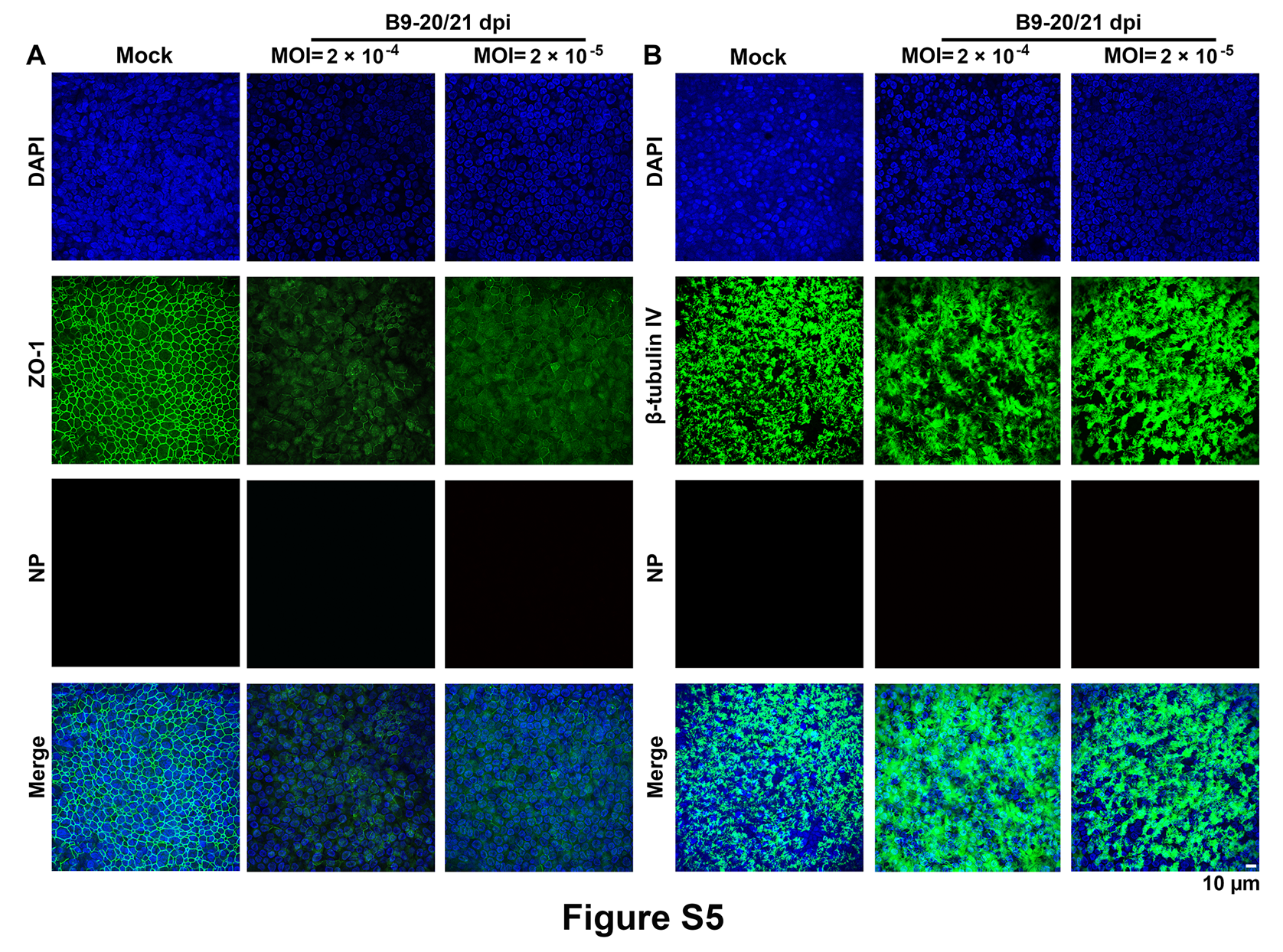

Supplement: FIG S5 [file mBio.02852-20-sf005.tif]

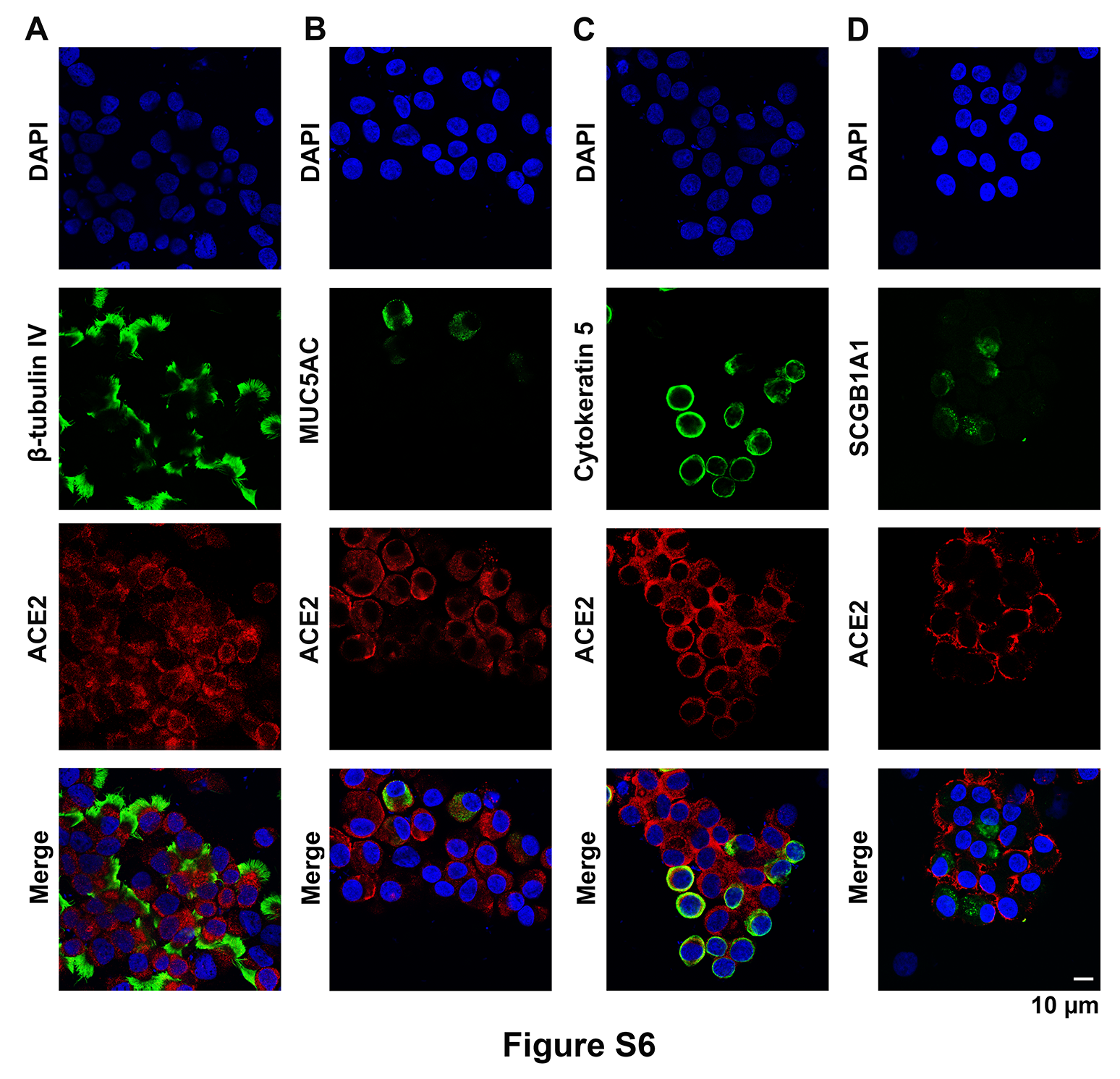

Supplement: FIG S6 [file mBio.02852-20-sf006.tif]
